# Tailored modular assembly derived self-healing polythioureas with largely tunable properties covering plastics, elastomers and fibers

**DOI:** 10.1038/s41467-022-30364-x

**Published:** 2022-05-12

**Authors:** Yan Mei Li, Ze Ping Zhang, Min Zhi Rong, Ming Qiu Zhang

**Affiliations:** Key Laboratory for Polymeric Composite and Functional Materials of Ministry of Education, GD HPPC Lab, School of Chemistry, Sun Yat-sen University, Guangzhou, 510275 China

**Keywords:** Polymers, Mechanical properties, Polymer synthesis

## Abstract

To impart self-healing polymers largely adjustable dynamicity and mechanical performance, here we develop libraries of catalyst-free reversible polythioureas directly from commodity 1,4-phenylene diisothiocyanate and amines via facile click chemistry based modular assembly. By using the amine modules with various steric hindrances and flexibilities, the reversible thiourea units acquire triggering temperatures from room temperature to 120 °C. Accordingly, the derived self-healable, recyclable and controlled degradable dynamically crosslinked polythioureas can take effect within wide temperature range. Moreover, mechanical properties of the materials can be tuned covering plastics, elastomers and fibers using (i) different assemble modules or (ii) solid-state stretching. Particularly, unidirectional stretching leads to the record-high tensile strength of 266 MPa, while bidirectional stretching provides the materials with biaxial strengths up to over 120 MPa. The molecular mechanism and technological innovations discussed in this work may benefit promotion and application of self-healing polymers towards greatly diverse demands and scenarios.

## Abstract (web summary)

Intrinsic self-healing polymers attract increasing attention but often suffer from a narrow self-healing temperature range and unsatisfactory mechanical performance. Here, the authors use click chemistry to develop a library of catalyst-free reversible polythioureas and demonstrate that the self-healing temperature and mechanical properties can be adjusted by controlling the flexibility and the steric environment.

## Introduction

Intrinsic self-healing polymers, which operate via reversible intra- and/or inter-macromolecular interactions^1^, have attracted increasing research attentions owing to their capability of autonomically recovering damages generated during manufacturing and usage^2,3^. So far, however, the adjustable range of self-healing temperature is quite narrow due to the restriction of reversible bonds themselves, such as the dissociation energy is difficult to be changed, which is not conducive to the expansion of working scenarios. The same family of dynamic reversible polymers is hard to serve as plastics, rubber and fiber at the same time. Besides, mechanical properties of the reported self-healing polymers can hardly reach the level of commodity polymers or engineering plastics^4–6^, mainly because of the lower bond dissociation energy (BDE) of reversible bonds^7^, and their mismatches with macromolecule skeletons and aggregation structure as well. Finally, the dynamic reversible moieties that are responsible for functioning of the smart materials used to be synthesized from relatively high cost chemicals by sophisticated processes^8–14^. As a result, popularization and application of intrinsic self-healing polymers have to be limited.

**Fig. 1:**
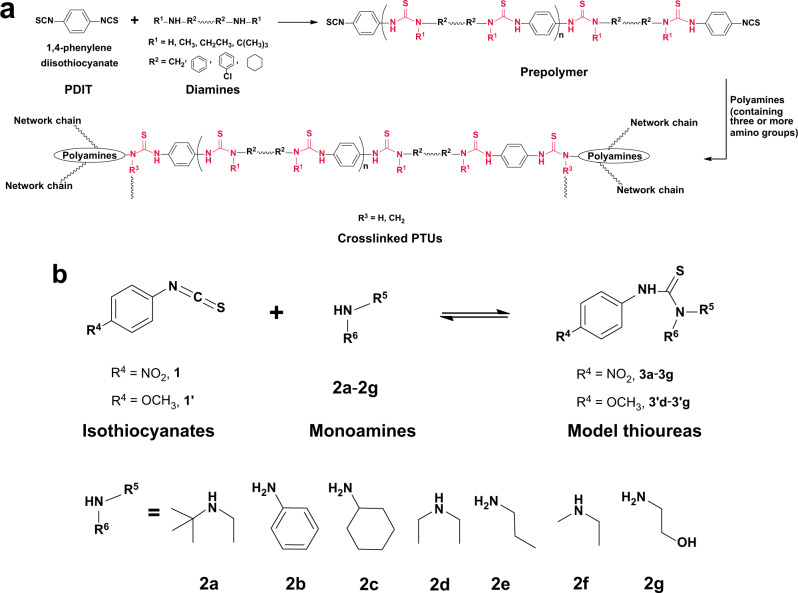
Design of polythiourea networks. **a** Synthesis of crosslinked PTUs containing thioureas. **b** Dissociation/association equilibriums of thioureas, and chemical structures of commercial isothiocyanates and monoamines.

**Fig. 2:**
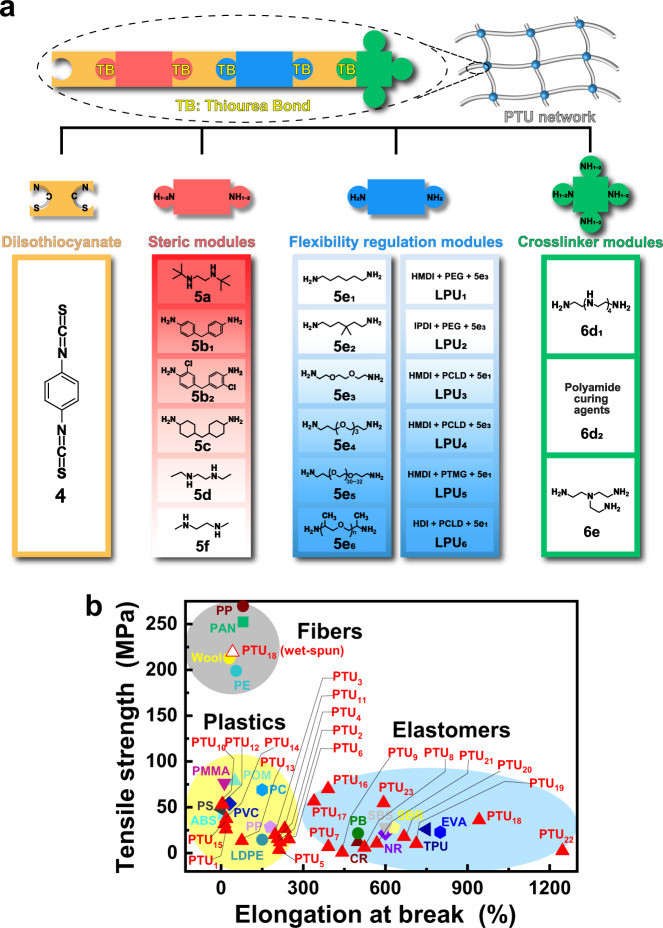
Synthesis and mechanical characterization. **a** Modular assembly-like synthesis of crosslinked PTUs, and chemical structures of the designed modules. Note: The darker background colors of the boxes for steric modules and flexibility regulation modules indicate larger steric hindrance, and greater ability of raising macromolecules’ flexibility, respectively. **b** Comparison of tensile properties of the crosslinked PTUs developed in this work with those of commodity plastics, elastomers and fibers. Note: ABS acrylonitrile butadiene styrene, CR chloroprene rubber, EVA ethylene vinyl acetate copolymer, LDPE low density polyethylene, NR natural rubber, PAN polyacrylonitrile fiber, PB polybutadiene, PC polycarbonate, PE polyethylene fiber, PMMA polymethylmethacrylate, POM polyformaldehyde, PP polypropylene, PS polystyrene, PVC polyvinyl chloride, SBR styrene butadiene rubber, SBS styrenic block copolymers, TPU thermoplastic polyurethane.

Here, we tackle the challenge by preparing crosslinked polythioureas (PTUs) containing thiourea bonds as reversible units from commodity isothiocyanates and amines (Figs. 1 and 2a) through a facile modular assembly-like method. The reaction between the chemicals that constitute the desired reversible bonds directly forms covalent adaptive networks, which is much simpler than most cases in the field reported so far^15–19^. By using such a modular assembly approach, molecular chain skeletons of the polymers and their properties can be easily adjusted. The C−N bond of thiourea unit in polythioureas^20^ is allowed to be involved in catalyst-free dissociation/association owing to the weak electron-withdrawing tendency of sulfur atom that diminishes the p-π conjugate between the lone pair electrons of N atom and π-electrons of C = S bond, and the sterically hindered effect of substituents^21^. Meantime, a wide variety of isothiocyanates and amines form the raw material basis of obtaining diverse thiourea bonds on demand in terms of click chemistry (also known as combinatorial chemistry), characterized by high efficiency, catalyst-free, oxygen/water insensitivity, atom-economic, and easy to be scaled up^22,23^. Under the circumstances, the dynamic reversibility (reflected by self-healing temperature) of the target PTU networks bearing thiourea units (including BDE of the latter) can be largely tuned by taking advantage of different steric hindrance effects of amines. Besides, type of crosslinkers, inter-molecular hydrogen bonding, crystallines and flexible chains provide rich measures for the wide range regulation of mechanical properties, while stretching induced topological rearrangement of the reversibly crosslinked PTU would further increase the strength. Hereinafter, structure-properties relationship of PTUs is carefully characterized and discussed. It is hoped that the opportunities offered by dynamic thiourea units can promote exploration of the smart materials and their practical applications.

## Results

### Dynamic reversibility of thioureas

At the beginning, the bond dissociation energies (BDEs) of C−N bonds in thioureas (i.e., aliphatic thioureas (I) and aromatic thioureas (II)) are estimated by the density functional method^24^ and compared with those of urea groups (III)^21^ (Supplementary Table 1). Thioureas possess lower BDEs than urea groups owing to the replacement of oxygen by sulfur. Moreover, BDE values of thioureas increase with decreasing the steric hindrance, which allows for the subsequent design and adjustment of dynamicity of thiourea bonds.

To further understand thermodynamic and kinetic characteristics of thioureas, typical isothiocyanate (4-nitrophenyl isothiocyanate (**1**) or 4-methoxyphenyl isothiocyanate (**1′**)) and monoamines (**2a**–**2g**) are employed to create a series of model thioureas (**3a**–**3g**, **3′d**–**3′g**, Fig. 1b). Their successful synthesis is confirmed by the Fourier transform infrared spectroscopy (FTIR), proton nuclear magnetic resonance (^1^H NMR), carbon-13 magnetic resonance (^13^C NMR) and elemental analysis (Supplementary Fig. 1 and Supplementary Information 1.2), except that the bulky steric hindrance of tertiary butyl in **2a** leads to coexistence of the raw chemicals and product **3a** at room temperature (Supplementary Fig. 1a).

The in-situ variable-temperature ^1^H NMR spectra (Supplementary Fig. 2) reveal that the association/dissociation equilibriums of the thioureas are temperature dependent, so that their dissociation temperatures, *T*_*d*_ (defined as the temperature at which signals of the dissociative products are able to be detected by in-situ variable-temperature ^1^H NMR within 30 min), and dissociation constants, *K*_*d*_, are obtained. Supplementary Table 2 exhibits that the *T*_*d*_ values of different thioureas gradually increase from room temperature to above 100 °C, whose reliance on the substituents’ steric hindrance agrees with that of BDE. Besides, *K*_*d*_ is enhanced with increasing not only temperature but also steric effect under a given temperature. Unlike the typical dissociative-type reversible Diels-Alder bonds (*K*_*d*_ ≈ 1.7 M at 120 °C^17^), dynamic thiourea chemistry possesses much smaller *K*_*d*_ in most cases, which would ensure structural integrity of the polymers bearing thiourea bonds at elevated temperature. Therefore, selection of thiourea with appropriate dissociation constant may help to balance dynamicity and mechanical properties of the materials.

On the other hand, the dissociation reactions are found to be endothermic (*ΔH*_*d*_ > 0) and entropy increase (*ΔS*_*d*_ > 0), as determined from the slopes and intercepts of *K*_*d*_ against temperature curves based on van’t Hoff equation (Supplementary Fig. 2f, Supplementary Table 2). The kinetics studies of thioureas based on in-situ variable-temperature ^1^H NMR spectroscopy further indicate that thermal dissociation of thiourea follows the first order kinetics (Supplementary Fig. 3). By fitting of the dissociation rate constant, *k*_*d*_, according to Arrhenius equation (Supplementary Fig. 3f), steric hindrance-dependent activation energies of dissociation, *E*_*a,d*_, are estimated. Clearly, the orders of magnitude range of these thermodynamic and kinetic parameters (Supplementary Table 2) cover those of most of the previously reported reversible covalent reactions^8^.

Based on the reversibly association/dissociation equilibrium of thioureas, the exchange kinetics between thioureas and amines are characterized using ^1^H NMR. Interestingly, the exchange temperatures of thioureas are significantly lower than their *T*_*d*_ values in the presence of the monoamines. For example, the dissociation of thiourea **3e** occurs at ~100 °C, but **3e** can easily exchange with monoamine **2d** in CDCl_3_ at ~60 °C, exhibiting an exchange activation energy, *E*_*a,e*_, of 90.2 kJ mol^−1^ (Supplementary Table 3, Supplementary Fig. 4a, b). Moreover, the exchange between the thiourea with larger steric hindrance (**3d**) and the amine **2e** becomes easier than that between **3e** bearing the minimal steric hindrance and **2d**, and the corresponding *E*_*a,e*_ decreases to 53.9 kJ mol^−1^ (Supplementary Table 3, Supplementary Fig. 4c, d). Meantime, we find that stronger alkalinity of amine (**2f**, p*K*_*b*_ ≈ 3.06; **2e**, p*K*_*b*_ ≈ 3.36^25,26^) improves the nucleophilic activity that is critical for formation of the intermediates in the associative path (Supplementary Fig. 8a), and hence leads to lower *E*_*a,e*_ (29.8 kJ mol^−1^, Supplementary Table 3, Supplementary Fig. 4e, f). As for phenyl amine (**2b**) with the weakest alkalinity (p*K*_*b*_ ≈ 9.40), **3d** can hardly exchange with it under similar conditions (Supplementary Fig. 5).

Likewise, the exchange kinetics between thioureas are studied by high-performance liquid chromatography (HPLC). The replacement of electron-withdrawing nitro groups attached to the benzene rings of thioureas by the electron-donating methoxy moieties effectively decreases their *T*_*d*_ (Supplementary Table 2 vs Supplementary Figs. 6g and 7g). The amine intermediates dissociated from the thioureas containing methoxy moieties (e.g., **3′f** and **3′g**) at lower temperature theoretically further accelerate exchange of the thioureas bearing nitro groups (e.g., **3d** and **3e**) via a combination of reversible dissociative and associative pathways (Supplementary Fig. 8b). As a result, the exchange temperatures are lower than *T*_*d*_ values of thioureas with nitro groups. For example, the exchange of thioureas **3d** and **3′f** in acetonitrile occurs at 45 °C (Supplementary Fig. 6a–e), which is much lower than the *T*_*d*_ of **3d** itself. The same thing also happens with thioureas **3e** and **3′g** (Supplementary Fig. 7a–e*vs* Supplementary Table 2). Meanwhile, Arrhenius analyses of the above systems yield *E*_*a,e*_ values of 85.7 and 99.2 kJ mol^−1^, respectively (Supplementary Table 3, Supplementary Figs. 6f and 7f). The dependence of *E*_*a,e*_ on thioureas’ steric hindrance coincides with that of the exchange between thioureas and amines revealed above.

In sum, the exchange rates (*k*_*e*_, 0.0018–2.26 h^−1^) and exchange activation energies (*E*_*a,e*_, 29.8–99.2 kJ mol^−1^) of thioureas are found to rely on the dissociation/association kinetics^27,28^ of thiourea species, alkalinity of amine and temperature. Previous reports suggested that the exchange rate is acceptable when it reaches up to 10^−2^ order of magnitude at an appropriate temperature^17,21,29–31^. Therefore, the highly tunable exchange kinetics of thioureas should be able to offer dynamicity on a reasonable time scale.

### Modular assembly-like synthesis of crosslinked polythioureas

As shown in the Introduction, we plan to prepare the target networked polythioureas simply via the click reaction between isothiocyanates and amines (Fig. 1a). No other reactive groups are involved. In this context, the synthesis proceeds similar to modular assembly with isothiocyanates and amines as the modular components (Fig. 2a). The latter can be conveniently combined and interchanged, simultaneously producing different PTUs and the built-in reversible thiourea bonds. Accordingly, precise control of constitution of the resultant, i.e. the primary level of structural hierarchy of polymer, is enabled, which is hard to achieve with conventional polymerization approaches (such as random copolymerization or polycondensation of multiple reactive groups^32^, Supplementary Fig. 1l).

In practice, the isothiocyanate module represented by 1,4-phenylene diisothiocyanate (PDIT, **4**) and various steric modules (**5a, 5b**_**1**_**, 5b**_**2**_**, 5c, 5d** and **5f**), flexibility regulation modules (**5e**_**1**_–**5e**_**6**_, **LPU**_**1**_–**LPU**_**6**_) and crosslinker modules (**6d**_**1**_**, 6d**_**2**_**, 6e**) served by polyamines bearing identical reactive moieties (i.e., amino) are involved in construction of the crosslinked PTUs (Fig. 2a). The benzene ring of PDIT would enhance the dynamicity of thiourea and improve its reactivity^32^. Besides, the steric modules (i.e. **5a**, **5d** and **5f**, the secondary amines with different substituents; and **5b**_**1**_, **5b**_**2**_ and **5c**, the primary amines with phenyl or cyclohexyl groups) bring in diverse steric hindrances and favor the manipulation of reversibility of the PTUs. As for the flexibility regulation modules and crosslinker modules, they are responsible for adjusting mechanical properties of the PTUs. Here, **5e**_**1**_–**5e**_**6**_ are the primary amines carrying methylene or methine without steric hindrance and possessing weak ability to form secondary interaction. Their conformational change would effectively affect the equilibrium flexibility of the ultimate macromolecules^33^. The higher degrees of free intramolecular rotation of C-O bonds of **5e**_**3**_–**5e**_**6**_ than that of C-C bond of **5e**_**1**_, and the side methyl of **5e**_**2**_ and **5e**_**6**_ that decreases the interaction between polymer chains, determines different deformation abilities of PTUs. Meanwhile, **LPU**_**1**_–**LPU**_**6**_ (Supplementary Fig. 9a) are diamino terminated linear polyurethanes bearing plentiful hydrogen bonds (Supplementary Fig. 10a) and crystalline phases (**LPU**_**6**_, Supplementary Fig. 11a).

By adopting the idea of modularization, a series of crosslinked PTUs are yielded (Supplementary Table 4). As illustrated in Fig. 2b, mechanical properties of the PTUs span from plastics to elastomers and fibers depending on assembling of different modules. It can be seen that these assembled dynamic polymers exhibit mechanical properties similar to those of conventional commercially available polymers. When the flexibility regulation modules (**5e**_**1**_–**5e**_**6**_) are coupled with the steric modules, for instance, tensile strengths of PTUs are basically enhanced with weakening of the ability of the former to raise macromolecules’ flexibility and increasing steric hindrance of the latter (Supplementary Table 4). Typically, **PTU**_**1**_ assembled from **4**, **5d**, **5e**_**1**_ and **6d**_**1**_ behaves like a rigid plastic at ambient temperature (Supplementary Figs. 9b and 10b, and glass transition temperature, *T*_*g*_, = 65 °C, Supplementary Fig. 11b), with tensile strength and elongation at break of 26.1 MPa and 17.7%, respectively. When **5e**_**1**_ is replaced by **5e**_**5**_, however, an elastomer **PTU**_**7**_ with *T*_*g*_ and failure strain of ~42 °C and 391.5% is obtained (Supplementary Fig. 11c and Supplementary Table 4).

Meanwhile, as higher segment regularity allows for closer stacking of molecular segments, polymers are strengthened. Accordingly, the flexibility regulation module **5e**_**6**_ bearing dense methyl side group in **PTU**_**9**_ is changed to **5e**_**5**_ that has no side group, while the other modules (i.e., **4**, **5d** and **6d**_**1**_) remain the same. Tensile strength of the resultant **PTU**_**7**_ indeed increases from 0.71 MPa (of **PTU**_**9**_) to 6.8 MPa. On the other hand, superior strengthening and toughening can be simultaneously realized (**PTU**_**16**_–**PTU**_**18**_) after the incorporation of the flexibility regulation modules of **LPU**_**3**_, **LPU**_**5**_ and **LPU**_**6**_. By taking **PTU**_**16**_ as an example, which performs like an elastomer (*T*_*g*_ = ~30 °C, Supplementary Fig. 11e), exhibiting tensile strength of 70.0 MPa and elongation to break of 390.6% (Supplementary Table 4). In addition, **PTU**_**18**_ can even be wet spun to get fibers (Supplementary Fig. 13a, b), whose breaking strength is as high as 202.7 MPa. Relatively, tensile strengths and failure strains of **PTU**_**1**_, **PTU**_**5**_ and **PTU**_**6**_ containing the flexibility regulation modules belonging to another group (i.e. **5e**_**1**_, **5e**_**3**_ and **5e**_**4**_) are 3.73–26.1 MPa and 17.7–247.2%, respectively. Considering that the rest modules involved in the above PTUs (i.e. **4**, **5d** and **6d**_**1**_) keep unchanged, the significant differences in properties gain highlight the greater contribution of the linear polyurethanes based modules than the primary amines based modules under the circumstances. The former build up much more intermolecular hydrogen bonds (for **LPU**_**3**_, **LPU**_**5**_ and **LPU**_**6**_) and introduce crystallites as reinforcements (for **LPU**_**6**_).

With respect to the influence of type of the crosslinker modules, the crosslinker module **6d**_**2**_ (Supplementary Fig. 9c) with abundant short branches is conductive for improving mobility of the neighbor macromolecular chains, so that elongation to break of **PTU**_**3**_ and **PTU**_**4**_ (~200%) is remarkably higher than that of **PTU**_**1**_ (17.7%) crosslinked by the regularly structured **6d**_**1**_, in the case that the other modules (i.e., **4, 5d** and **5e**_**1**_) are fixed^34^.

### Self-healability of crosslinked PTUs

**Fig. 3:**
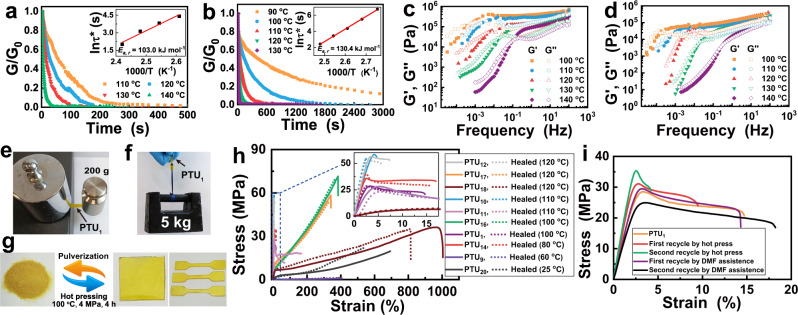
Dynamicity, self-healability and reprocessability. Normalized stress relaxation of (**a**) **PTU**_**1**_ and (**b**) **PTU**_**11**_ as a function of temperature. The insets show the temperature variations of the characteristic relaxation time, τ*, fitted according to Arrhenius equation. Storage shear modulus, *G’*, and loss shear modulus, *G”*, versus frequency of (**c**) **PTU**_**1**_ and (**d**) **PTU**_**11**_ recorded at different temperatures, respectively. Healed version of bisect **PTU**_**1**_ subjected to (**e**) a lateral load of 200 g in the single cantilever mode, and (**f**) a 5 kg weight in the parallel direction (35 × 2 × 1.2 mm). **g** Photos showing molding of pulverized **PTU**_**1**_ into square sheet and dog-bone-shaped specimens via hot pressing (100 °C, 4 MPa, 4 h). **h** Typical tensile stress-strain curves of the virgin and repaired PTUs. **i** Typical tensile stress-strain curves of the recycled **PTU**_**1**_.

In the preceding discussion thioureas prove to be thermally reversible and the reversibility can be easily designed and regulated, which has been factually inherited by the PTUs (Supplementary Fig. 14). As shown by the gel permeation chromatography (GPC) measurements, reshuffling of linear PTUs is allowed under certain temperatures (Supplementary Fig. 15). The molecular weights of the exchange products are in the middle of those of the original linear PTUs. More importantly, stress relaxation behaviors of typical crosslinked PTUs (i.e. **PTU**_**1**_ and **PTU**_**11**_) also confirm their dynamicity. That is, both PTUs are able to relax stress to zero at certain temperature, and faster relaxation is observed at higher temperature on account of the acceleration of thiourea exchange kinetics (Fig. 3a, b).

Since temperature dependences of the characteristic relaxation time, τ* (determined at 1/e of the normalized stress relaxation) according to Maxwell law^35^, are found to obey Arrhenius equation, the relaxation activation energy, *E*_*a,r*_, of **PTU**_**1**_ is calculated to be 103.0 kJ mol^−1^ accordingly, which is lower than those of **PTU**_**11**_ (130.4 kJ mol^−1^) and the thiourea network containing aliphatic hindered thiourea bonds reported by Feng et al. (115.8 kJ mol^−1^)^36^ owing to the greater dynamicity of the thiourea units in **PTU**_**1**_ ascribed to the **5d** module and aromatic PDIT (**4**), respectively. The result agrees with not only our study of small molecules at the beginning, but also the correlation between relaxation of crosslinked networks and steric hindrance of reversible bond reported elsewhere^37^. In fact, **PTU**_**1**_ includes smaller steric hindrance module (**5d**) and **PTU**_**11**_ doesn’t contain any steric hindrance module. They are representative enough for other PTUs prepared in this work. In sharp contrast, the control polyurea excluding dynamic thiourea bonds (Supplementary Fig. 9d) doesn’t exhibit decent reversibility under the same conditions, as characterized by its steady stress relaxation manner analogous to irreversibly crosslinked thermosets (Supplementary Fig. 16).

The frequency dependences of storage modulus, *G’*, and loss modulus, *G”*, of **PTU**_**1**_ and **PTU**_**11**_ at various temperatures (Fig. 3c, d) show that *G’* is higher than *G”* over a wide range of frequency like typical viscoelastic solid (irreversibly crosslinked networks), but the order is reversed in the low frequency regime presenting a viscous behavior (*G”* > *G’*), and the resultant dissociated networks require ample time to be reestablished. It is interesting to see that the apparent terminal relaxation time, *τ*_*d*_^3,38^, which reflects lifetime of reversible bonds in the macromolecules calculated from reciprocal of the crossover frequency, is closely relevant to the dissociation rate constant, *k*_*d*_, of thiourea (Supplementary Fig. 3d, Supplementary Fig. 3e, and Supplementary Table 2). Higher temperature raises *k*_*d*_ and causes smaller *τ*_*d*_. Besides, *τ*_*d*_ values of **PTU**_**1**_ (7.9–1584.8 s) are smaller than those of **PTU**_**11**_ (10.0–6468.3 s) at a given temperature owing to the higher *k*_*d*_ of the built-in thiourea units with higher steric hindrances. Other groups also revealed that *τ*_*d*_ of reversibly crosslinked polymers ranged from a few seconds to thousands of seconds^38,39^. The appropriate relaxation time scale provides necessary viscoelasticity for implement of solid-state reversible reaction at the crack interface, while the wide range of relaxation times means that these materials can be easily adjusted in terms of healing time and temperature.

**Fig. 4:**
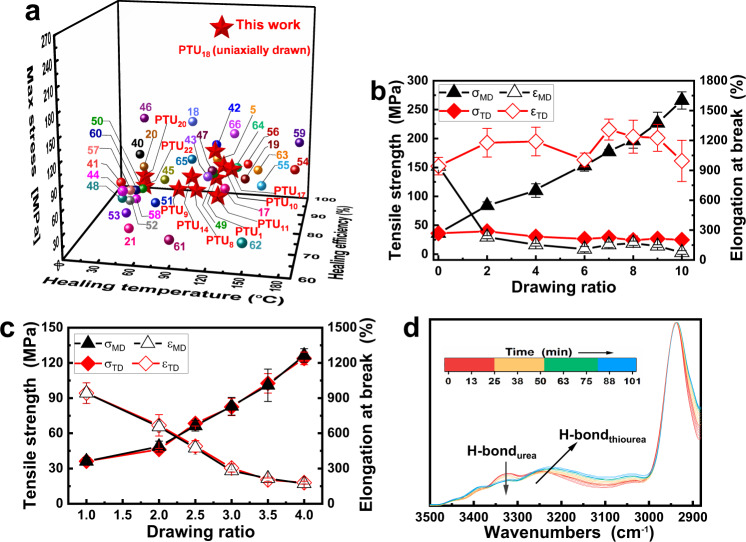
Dynamicity and mechanical properties. **a** Relationships between tensile strength and self-healing efficiency and trigger temperature of various PTUs in comparison with those of the previously reported healable polymers containing different reversible covalent/non-covalent bonds (The numbers next to symbols represent the bibliographies. Here, the strengths refer to those of the original materials. All the materials are not treated by solid-state drawing, except **PTU**_**18**_ that is stretched to the maximum stroke (1 mm min^−1^, 60 °C) and kept for 100 min). **b**, **c** Tensile strengths and elongation at break of (**b**) uniaxially and (**c**) biaxially drawn **PTU**_**18**_ as a function of drawing ratio (The error bars show the standard deviations from at least five tests). **d** Real-time FTIR spectra of the -NH- peaks of stretched **PTU**_**18**_.

By taking advantage of the dynamic characteristics, the crosslinked PTUs are allowed to be self-healed and reprocessed. Firstly, thermally induced crack healing of **PTU**_**1**_ and **PTU**_**11**_ are evidenced by visual inspection (Supplementary Fig. 17a, b). Meanwhile, the recombined bisect **PTU**_**1**_ can lift a lateral load of 200 g in single cantilever mode (Fig. 3e), and a weight up to 5 kg (~3 × 10^4^ times its own weight, Fig. 3f). Next, tensile tests are conducted to quantitatively evaluate healing efficiencies of the PTUs. Despite the linear polythiourea with self-healability based on hydrogen bonds^20^, to our knowledge, few reports deal with dynamic reversibility of thiourea bonds^36^. Figure 3h and Supplementary Table 4 illustrate that self-healing of the crosslinked PTUs can proceed between room temperature and 120 °C with healing efficiencies mostly higher than 90%, and their mechanical strengths are at the forefront of the reported self-healable materials (Fig. 4a)^5,17–21,40–66^. What is more, a wide variety of self-healable PTUs only based on single reversible thiourea bond can possess properties comparable to previous dynamic polymers made from different formulas, methods and reversible bonds. This potentially fits the recycling requirement of simplifying the types of materials in practice.

To achieve high self-healing efficiency, matching among healing temperature, reversible thermodynamics and kinetics, and macromolecular chain mobility should be guaranteed. In particular, healing temperature should be higher than the exchange temperature of thioureas, and *T*_*g*_ of the polymer to be repaired. As viewed from practical usage, self-healing at a temperature above room temperature would be more desirable because the unwanted creep under ambient conditions can thus be prevented. Moreover, self-healing temperature had better to be lower than the working temperature of the materials to avoid structural instability during service. For intrinsic self-healing polymers, therefore, a broad tunability of self-healing temperature is as important as the mechanical performance, and the widely available reactive modules of thioureas can precisely meet the demand.

As shown in Supplementary Table 4, healing temperature (defined as the minimum empirical healing temperature achieving a healing efficiency above 80% within 24 h) of the crosslinked PTUs can be adjusted by purposely assembling different modules. For the PTUs containing thiourea **3d**, not only the tough **PTU**_**16**_ and **PTU**_**17**_ realize satisfactory healing at 100 °C and 120 °C (Figs. 3h and 4a), respectively, but also the rigid **PTU**_**1**_ and **PTU**_**10**_ can restore 98% and 94% of strength at about 100 °C. Accordingly, thiourea bond **3e**-bearing **PTU**_**11**_ shows 87% strength retention after thermal treatment at 110 °C. Even though the dissociation temperature of thiourea **3c** is lower than those of **3d** and **3e**, healing of the corresponding **PTU**_**12**_ is enabled at 120 °C owing to its highest *T*_*g*_ (~100 °C, Supplementary Fig. 11d) giving 92% strength recovery. As for the thioureas (**3a** and **3b**) with higher dynamicity than **3c**, the derived PTUs are able to restore mechanical properties at lower temperature. For example, the rigid **PTU**_**14**_ is allowed to be healed at 80 °C with healing efficiency of 93%, while 84–100% of strengths of elastomers **PTU**_**19**_–**PTU**_**22**_ (Fig. 3h, Supplementary Table 4) can be regained at room temperature. On the contrary, the control polyurea fails to repair crack and recover mechanical properties under similar conditions (Supplementary Fig. 17c, d).

In addition to thiourea bonds, other modules also affect healing temperature. For the PTUs consisting of the flexibility regulation modules of linear polyurethanes (**LPU**_**1**_–**LPU**_**6**_, Supplementary Fig. 9a), whose glass transition temperatures are lower than room temperature (Supplementary Figs. 11e–j and 12), the healing temperature decreases with increasing the steric hindrance of the steric modules (e.g., the healing temperatures of **PTU**_**16**_–**PTU**_**18**_ containing **5d** is higher than 100 °C, whereas **PTU**_**19**_–**PTU**_**22**_ carrying **5a** or **5b**_**1**_ are healable at room temperature). When the steric module is fixed (e.g., **5d**), the healing temperatures of **PTU**_**1**_ and **PTU**_**5**_–**PTU**_**9**_ (100–60 °C) decrease with increasing the ability of raising macromolecules’ flexibility of the flexibility regulation modules (from **5e**_**1**_ to **5e**_**6**_).

It can thus be concluded that increased steric hindrance of thiourea bond and chain flexibility as well are advantageous to reduce healing temperature of PTU_S_, and verse vice.

Besides, solid-state recycling and solution-assisted degradation/regeneration of the crosslinked PTUs are demonstrated (Fig. 3g and Supplementary Fig. 18a). The hot pressed sheet from pulverized **PTU**_**1**_ has similar mechanical properties as the virgin one, and the recycling can be repeatedly conducted (Fig. 3i). Simultaneously, the controllable degradation of **PTU**_**1**_ is achieved by immersing the specimen in N,N-dimethylformamide (DMF) containing different contents of amine **5d** (0.001–0.1 mol·L^–1^) under different temperatures (30–60 °C) for different times. Higher temperature and amine concentration shorten the degradation time from 500 to 0.5 h (Supplementary Fig. 18b). Number average molecular weights (*M*_*n*_, 7790–15932 g mol^−1^) of the degraded products decrease with a rise of amine content (Supplementary Fig. 18c), which are nearly independent of temperature because exchange reaction predominates in this case. Notably, the degraded products are able to be re-polymerized by adding equivalent mole of isothiocyanate **4** and reacting for 24 h. The regenerated materials show insignificant attenuation of mechanical performance (Fig. 3i). In fact, the addition of amines that are different from the amines in the PTUs can regulate the molecular structure and properties of the latter.

### Uniaxial and biaxial strengthening via solid-state drawing

It has been known that crosslinked polythioureas can be imparted with wide range of mechanical properties and self-healing capability by assembling proper modules (Figs. 2 and 3). In fact, the dynamic thioureas can make even greater contribution, for example, allowing the networked materials to be further strengthened through solid-state stretching.

Unlike the irreversibly crosslinked polymers that cannot be extended after curing, the macromolecular chains of dynamic crosslinked networks are able to orient along the direction of external force under the assistance of breaking/recombination reactions of reversible bonds^18,67^. Therefore, the amount of effective load bearers increases. The dissociation of the reversible bonds is equivalent to temporarily de-crosslinking partial networks and removing the restriction that prohibits extension of the molecular chains. Following the stretching process, reconnection of the dissociated reversible bonds reforms the networks with extended segments to get the best of both thermoplastics and thermosets. Figure 4b, c illustrates this is true. Tensile strength of crosslinked **PTU**_**18**_ is raised not only in one direction, but also in both directions which are perpendicular to each other, after uniaxial and biaxial drawing (at 60 °C and 105 °C, respectively) above the reversible temperature of the built-in thiourea unit **3d** (45 °C). The key factor, i.e. dynamicity of crosslinked **PTU**_**18**_ under the circumstances, is verified by the relaxation behaviors (Supplementary Fig. 19). The typical stress-strain curves of the oriented **PTU**_**18**_ (Supplementary Fig. 20) further reveal that the values of strength and elongation at break also reach the levels of plastics, elastomers and fibers (Supplementary Fig. 21) depending on the drawing ratio. One thing needs to be mentioned is that the crystalline polycaprolactone segments (*T*_*m*_ = 47.4 °C, Supplementary Fig. 11f) included in **PTU**_**18**_ have to be melted during the stretching, but macroscopic collapse of the material doesn’t occur. Because of dynamic equilibrium of the dissociation/reconnection of thioureas and the much smaller *K*_*d*_ than that of DA bond, **PTU**_**18**_ is in the rubbery plateau at the drawing temperatures (Supplementary Fig. 12).

In the case of uniaxial stretching (Fig. 4b), tensile strength along the drawing direction (i.e. machine direction (MD)), *σ*_*MD*_, of **PTU**_**18**_ increases with increasing drawing ratio and reaches the maximum of 265.88 MPa at the drawing ratio of 10. Wide-angle X-ray diffraction (WXRD) spectroscopic study indicates that the reinforcement keeps pace with the enhancement of crystalline orientation degree (0.423–0.544) (Supplementary Fig. 22a and Supplementary Table 5)^68^. The finding is supported by the results of polarizing optical microscope observation, which confirm the obvious alignment of crystalline regions in the uniaxially drawn **PTU**_**18**_ under orthogonally polarized light (Supplementary Fig. 23e–h). Interestingly, the strength in the perpendicular direction (i.e. transverse direction (TD)), *σ*_*TD*_, remains nearly unchanged, which is different from conventional oriented thermoplastics (i.e. increase of *σ*_*MD*_ used to be accompanied by the great attenuation of *σ*_*TD*_^69^). The synchronous recombination of reversible crosslinks in the perpendicular direction should account for this special feature. With respect to the failure strain along the drawing direction, *ε*_*MD*_, it inevitably decreases initially with the drawing ratio and then remains essentially constant, but in the vertical direction, *ε*_*TD*_ is also independent of drawing ratio. Moreover, the **PTU**_**18**_ that suffers from cutting/healing can be drawn again (Supplementary Fig. 24), leading to *σ*_*MD*_ of 203.1 MPa along the parallel direction. It is thus known that this approach offers a solution to address the dilemmas in self-healable materials that high mechanical robustness and healing efficiency are mutually exclusive.

When biaxial drawing is concerned (Fig. 4c), the tensile strengths of **PTU**_**18**_ along two vertical directions are synchronously improved, but the reinforcing effect is weaker than those of uniaxially oriented materials, owing to lower planar orientation of the macromolecular chains as verified by the nearly isotropic reflections in the two-dimensional WXRD image (Supplementary Fig. 22b–d)^70^. Typically, the *σ*_*MD*_ and *σ*_*TD*_ values increase to 123.8 MPa and 126.3 MPa at the drawing ratio of 4, respectively, which are comparable to commercial biaxially oriented plastics (e.g., PE, 69–127 MPa; polylactic acid (PLA), ~110 MPa; PS, 60–80 MPa). Under the circumstances, *ε*_*MD*_ and *ε*_*TD*_ are still 179.3 % and 171.2%. The crosslinked polymer is thus isotropically strengthened, and the intractable anisotropy originating from uniaxial solid-state drawing is solved at the same time.

It is worth noting that not all reversibly crosslinked polymers are bound to be well strengthened by solid-state drawing. We believe that at least two conditions need to be satisfied. (i) The macromolecular chains should be long enough to provide the space for extension under stretching. (ii) Relative sliding of the macromolecular chains is enabled for regular accommodation of the latter along the direction of external force.

In the case of **PTU**_**8**_ and **PTU**_**12**_, for example, the number average molecular weights of their linear prepolymers, *M*_*n*_, are 16184 g mol^−1^ and 47069 g mol^−1^, respectively, which are much lower than that of **PTU**_**18**_ (*M*_*n*_ ~ 200647 g mol^−1^). Consequently, they can only be slightly strengthened with marginal enhancement ratios of 1.02 and 1.27, respectively (Supplementary Table 6). On the other hand, *M*_*n*_ values of the prepolymers of **PTU**_**17**_ and **PTU**_**23**_ (Supplementary Table 6) are 147995 g mol^−1^ and 142137 g mol^−1^, respectively. Their molecular chains can be greatly extended, so that the enhancement ratios become ~2 (Supplementary Table 6). Nevertheless, their orientation performance is still not as satisfactory as that of **PTU**_**18**_, due to the incorporation of rigid 4,4’-methylenebis(cyclohexyl isocyanate) (HMDI) in the soft segments.

Additionally, in-situ variable temperature FTIR spectra (Fig. 4d) demonstrate the sliding of micromolecular chains of **PTU**_**18**_ during drawing. The peak intensity of the -NH- groups on the urea units decreases with stretching, reflecting dissociation of the urea-based hydrogen bonds when the curling polyurethane segments gradually extend. By contrary, the peak of the -NH- groups on the thiourea units significantly shifts towards lower wavenumber (from 3196 to 3181 cm^−1^), which indicates that more linear thiourea-based hydrogen bonds are formed during rearrangement of the macromolecular chains (Supplementary Fig. 25a, b). The variations (Fig. 4d) confirm that the macromolecular chains have slipped to a more ordered position under the applied force. Eventually, closure of the reversible bonds and crystallization of the melted crystals with the removal of thermal stimulus freeze the drawn state of the macromolecular chains (Supplementary Fig. 25b). The similar changes of H-bonds also happen in **PTU**_**23**_ (Supplementary Fig. 25c). As for **PTU**_**8**_, however, the inconspicuous sliding of the short molecular chains leads to negligible change of hydrogen bonds (Supplementary Fig. 25d). The improvement of its strength after solid-state stretching has to be insignificant (Supplementary Table 6).

## Discussion

This work investigates the dynamic reversibility of thiourea bonds, and constructs a variety of dynamically reversible crosslinked polythioureas with properties comparable to plastics, rubbers and fibers^42,43,49,68^ through the modular assembly-like synthesis. The designed thiourea bonds prove to be qualified for acting as the reversible units capable of participating the modular assembly and tuning the dynamicity within a wide temperature range by simply using amines with different steric hindrances. The preparation of the crosslinked polythioureas based on thiourea chemistry overcomes the deficiencies of the existing methods for making intrinsic self-healing polymers^8–19^, exhibiting unique advantages of high efficiency, catalyst free, atomic economy, wide scope of monomers, easy to proceed and scale up. The dynamic thiourea bonds present a number of desirable properties for dynamic polymers, like self-healability, solid-state recyclability, controlled degradability, and tunable mechanical properties.

More importantly, the utilization of single thiourea bond to connect different modular units forming polythioureas facilitates not only regulation of self-healing temperature, but also synergy between the dynamicity of the reversible bonds and the macromolecular backbones, thus achieving a reconciliation between self-healing habit and mechanical properties of the material. In addition, tensile strengths of the crosslinked polythioureas can be substantially increased by unidirectional or bidirectional stretching by taking effect of reversibility of the thiourea bond, which is an important step toward practical application. It is anticipated that the dynamic chemistry of thioureas can be readily integrated with the design of other polymers, bringing in refreshing properties, and the technique developed by the authors have sufficiently broad expandability.

## Methods

### Materials

4-Nitrophenyl isothiocyanate (**1**, 98%), 4-methoxyphenyl isothiocyanate (**1′**, 98%), N-tert-butylethylamine (**2a**, 98%), aniline (**2b**, 99%), cyclohexylamine (**2c**, 99%), diethylamine (**2d**, 99.5%), propylamine (**2e**, 98%), N-ethylmethylamine (**2f**, 98%), ethanolamine (**2g**, 99%), 4,4′-methylene-bis(2-chloroaniline) (**5b**_**2**_, 98%), trimethylhexamethylenediamine (**5e**_**2**_, 99%), polyethylene glycol bis(3-aminopropyl) ether (**5e**_**5**_, *M*_*n*_ = 1500 g mol^−1^), N,N’-diethylethylenediamine (**5d**, 95%), pentaethylenehexamine (**6d**_**1**_, mixture) and tris(2-aminoethyl)amine (**6e**, 97%) were purchased from Tokyo Chemical Industry Co., Ltd, Japan. 1,4-Phenylene diisothiocyanate (PDIT, **4**, 98%), phenyl isothiocyanate (PITC, 98%), N,N’-di-tert-butylethylenediamine (**5a**, 98%), 4,4’-methylenedianiline (**5b**_**1**_, 98%), di(p-aminocyclohexyl)methane (**5c**, mixture of isomers, 97%), hexane-1,6-diamine (**5e**_**1**_, 99.5%), 1,8-diamino-3,6-dioxaoctane (**5e**_**3**_, 98%), diethylene glycol bis(3-aminopropyl) ether (**5e**_**4**_, 98%), N,N’-dimethylethylenediamine (**5f**, 97%), 4,4’-methylenebis(cyclohexyl isocyanate) (HMDI, mixture of isomers, 99%), hexamethylene diisocyanate (HDI, 99%), isophorone diisocyanate (IPDI, 99%), m-xylylene diisocyanate (**4′**, XDI, 98%), N,N-dimethylformamide (DMF, 99.9%), acetonitrile (MeCN, 99.9%), methanol (MeOH, 99.5%) and dichloromethane (DCM, 99%) were supplied by Aladdin, China. Polyamide curing agents (**6d**_**2**_, 125: 35000 mPa·s at 25 °C, 260 mgKOH g^−1^; 115: 36000 mPa·s at 40 °C, 190 mgKOH g^−1^) were purchased from Wuhan Jofengyun New Materials Co. Ltd. Poly(propylene glycol) bis(2-aminopropyl ether) (PEA, **5e**_**6**_, *M*_*n*_ = 400 and 900 g mol^−1^), polycaprolactone diol (PCLD, *M*_*n*_ = 1000 and 3000 g mol^−1^), poly(ethylene glycol) (PEG, *M*_*n*_ = 400 and 600 g mol^−1^), and polytetramethylene ether glycol (PTMG, *M*_*n*_ = 1000 g mol^−1^) were purchased from Sigma-Aldrich. PCLD, PEA, PEG and PTMG were dehydrated at 100 °C under vacuum for more than 12 h prior to use. N,N-dimethylformamide (DMF, 99.9%) was dried by activated molecular sieves (4 Å) before usage. All the other chemicals and solvents were used as received.

**Syntheses of PTUs. PTU**_**1**_ was prepared by slowly adding the mixture of **5d** (1.04 g, 8.4 mmol) and **5e**_**1**_ (1.62 g, 13.65 mmol) into the DMF solution (60.0 mL) of 1,4-diisothiocyanatobenzene (**4**, 5 g, 25.2 mmol) in the nitrogen atmosphere. The system was stirred overnight at 60 °C, and then reacted with **6d**_**1**_ (0.25 g, 1.05 mmol) for 12 h. Finally, the mixture was poured into a silicone mold, and then cured at 60 °C for 48 h and thoroughly dried to obtain **PTU**_**1**_. **PTU**_**2**_–**PTU**_**10**_ were prepared in the same way.

As for **PTU**_**11**_, it was synthesized following the above steps, except that the mixture of **5d** and **5e**_**1**_ was replaced by 10.5 equiv. of **5e**_**4**_. The syntheses of **PTU**_**12**_–**PTU**_**15**_ referred to **PTU**_**11**_.

**PTU**_**18**_ was produced through solution polymerization. The dehydrated PCLD (3000 g mol^−1^, 19.5 g, 6.5 mmol) was stirred at 80 °C. Then, HDI (2.21 g, 13 mmol) and 3 drops of DBTDL were added. After 3 h, the system was cooled down to 60 °C, and **5e**_**1**_ (1.53 g, 13 mmol) containing 100 mL DMF was slowly dropped into the mixed solution, followed by stirring in argon for 4 h to obtain **LPU**_**6**_. Afterwards, the **LPU**_**6**_ solution continued to react with **4** (2.37 g, 12 mmol) and **5d** (0.49 g, 4 mmol) for additional 12 h. At last, the mixture was crosslinked by **6d**_**1**_ (0.12 g, 0.5 mmol), and then poured into a silicone mold, and vacuum dried to obtain **PTU**_**18**_ for the subsequent characterization. **PTU**_**16**_, **PTU**_**17**_ and **PTU**_**19**_–**PTU**_**23**_ were synthesized following similar procedures.

### General characterizations

Proton nuclear magnetic resonance (^1^H NMR) and carbon-13 magnetic resonance (^13^C NMR) spectra were recorded on a Bruker Advance III spectrometer. Elemental analyses were performed by an Elemental Analyzer (Vario EL). Fourier transform infrared spectra (FTIR) were recorded on a Nicolet Nexus 670 FTIR spectrophotometer. Number-average molar weight, *M*_*n*_, was determined at 50 °C by Waters Breeze gel permeation chromatography (GPC) system with DMF/LiBr solution (0.05 mol L^−1^ LiBr, 1 ml min^−1^) as eluent and polystyrene standards for calibration. Thermogravimetric analysis (TGA) was performed on a Thermogravimetry (TGA-Q50, Waters) under a nitrogen atmosphere at a heating rate of 10 °C min^−1^. Differential scanning calorimetry (DSC) measurements were conducted on a TA Instruments DSCQ10 using a nitrogen purge at a heating rate of 20 °C min^−1^. Tensile test was carried out at 25 °C by a SANSCMT6103 universal tester in accordance to ISO527-2 using dumbbell-shaped specimens (35 × 2 × 0.3–0.7 mm^3^), and each result was the average from at least five samples. The crystalline morphologies of the samples were observed by a polarizing optical microscope (Leica DM2700P).

### Stress relaxation and rheological tests

Stress relaxation studies were conducted on an ARES/RFS II Rheometer in parallel-plate configuration. The tests were performed in a strain control mode (1%) at specified temperatures. Activation energy of the exchange reaction, *E*_*a,r*_, was estimated from:

1 $${ln}\,{\tau }^{* }\,{{\mbox{=}}}\,{ln}\left(A\right)\,{{\mbox{+}}}\,\frac{{E}_{a,r}}{{RT}}$$

where τ^*^ is the characteristic relaxation time defined as the time required for *G*/*G*_*0*_ = 1/e; *G*_*0*_, *R* and *T* are the initial relaxation modulus, universal gas constant and absolute temperature, respectively.

Dynamic oscillatory frequency sweeps of modulus were carried out under the strain amplitude 1% at 60–140 °C from 100 to 0.0001 or 0.001 Hz by Kinexus pro+ Rheometer.

### Evaluation of self-healing effect

Qualitative evaluation of crack healing was conducted by visual inspection. **PTU**_**1**_, **PTU**_**11**_ and the control polyurea were firstly scratched by a sharp thin blade, and then repaired under certain conditions (80 °C, 2.5 h for **PTU**_**1**_, 100 °C, 6 h for **PTU**_**11**_ and 100 °C, 8 h for the polyurea). Changes of the cracks were recorded by a camera attached to a KEYENCE VHX1000C 3D optical microscope.

To quantitatively evaluate the healing effect, dumbbell-shaped specimens (35 × 2 × 0.3–0.7 mm^3^) were bisect, and then the two pieces were brought into contact for healing. Tensile tests were carried out to determine tensile strengths of the virgin and healed specimens, and healing efficiency is defined as the tensile strength ratio of the healed specimen to the virgin one.

### Solid-state drawing

Uniaxial solid-state drawing was conducted by a Hounsfield T10K-S universal tester equipped with a temperature-controlled cabinet. The rectangle-shaped specimens (60 × 45 × 0.5 mm^3^, grip to grip separation: 15 mm) were heated and then stretched to different draw ratios at a fixed speed (1 or 0.5 mm min^−1^, refer to Supplementary Table 6 for details of the drawing conditions). Finally, the specimens were slowly cooled down to 25 °C to obtain the drawn versions.

Biaxial synchronous drawing was performed by FOOL-100 Biaxial Extensometer at 105 °C under a speed of 20 mm s^−1^. The drawn specimens were moved to an incubator set at 60 °C for 0.5 h, and then cooled down to room temperature.

### X-ray diffraction (XRD) measurements

The crystalline structure of **PTU**_**18**_ was characterized by means of Phi-scan with Rigaku X-ray diffractometer (9 kW). The degree of orientation was estimated from:

2 $${{{\mbox{O}}}}_{{{\mbox{rel}}}}{{\mbox{=}}}1-\frac{{{\mbox{FWHM}}}}{{180}^{{{\mbox{o}}}}}$$

where *O*_*rel*_ is the relative orientation parameter, and FWHM is the full-width at half maximum of the diffraction peak fixed at the 2θ of 21.3°.

Two-dimensional (2D) wide-angle X-ray diffraction (WXRD) measurements were conducted on the X-ray source (maximum output power: 2.97 kW; electron beam focal spot diameter: 70 μm; Cu Kα) equipped with a HypiX-6000 photon direct reading detector.

### In-situ variable temperature FTIR spectroscopy study during solid-state drawing

The variations in hydrogen bonds during solid state drawing were measured by a Thermo Fisher DXR3xi micro-infrared spectrometer with an in-situ variable temperature stretching test bench (maximum stroke: ~67 mm). Firstly, the sample film was fitted to the stretching bench, and then heated to 60 °C for 5 min. Next, the sample film was stretched at a speed of 1 mm min^−1^, and the real-time spectra were recorded.

## Supplementary information

Supplementary Information

## Data Availability

All data supporting the findings of this study are available within the article and the Supplementary Information file. All data are available on request from the corresponding authors.
