# Supplementary material for: Tailored modular assembly derived self-healing polythioureas with largely tunable properties covering plastics, elastomers and fibers
Source: Nat Commun. 2022 May 12;13:2633. doi: 10.1038/s41467-022-30364-x (PMC9098433; doi:10.1038/s41467-022-30364-x)
Supplement: Supplementary file 1 — Supplementary Information [file 41467_2022_30364_MOESM1_ESM.pdf]

Supplementary Information

Tailored modular assembly derived self-healing polythioureas with largely tunable properties covering plastics, elastomers and fibers

Yan Mei Li[1], Ze Ping Zhang[1]*, Min Zhi Rong[1] and Ming Qiu Zhang[1]

[1] Key Laboratory for Polymeric Composite and Functional Materials of Ministry of Education, GD HPPC Lab, School of Chemistry, Sun Yat-sen University, Guangzhou 510275, China. *E-mail: zhangzp8@mail.sysu.edu.cn

1. Supplementary Methods

1.1 Calculations of Bond Dissociation Energies

Density functional method (revised Perdew-Burke-Ernzerhof functional derived in the generalized gradient approximation[1]) was used for the estimation.

Supplementary Table 1 Bond dissociation energies of thiourea and urea derivatives

C-N	BDE (kJ mol^{-1})	C-N	BDE (kJ mol^{-1})	C-N	BDE (kJ mol^{-1})
**Ia**	247.0	**IIa**	241.6	**IIIa**	279.8
**1b**	260.9	**IIb**	254.7	**IIIb**	301.8
**Ic**	261.7	**IIc**	255.1	**IIIc**	306.9
**Id**	298.5	**IId**	293.5	**IIId**	330.8

Note:

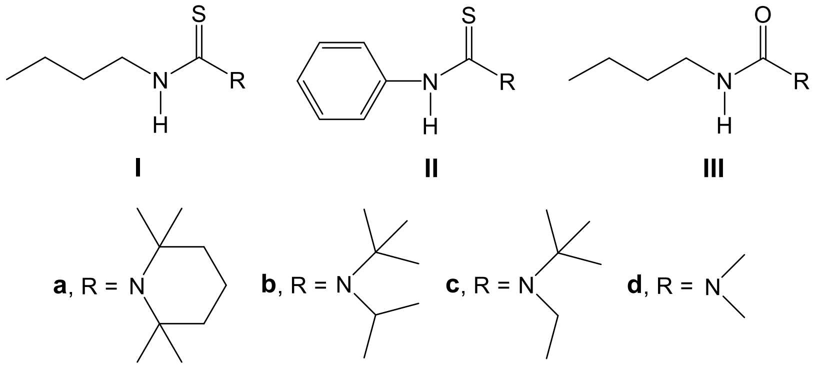

1.2 Synthesis of Model Thioureas

Synthesis of Model Thioureas 3a-3g. Thiourea compounds **3a**-**3g** were synthesized by mixing **1** (0.002 mol) with excessive **2a-2g** (0.0022 mol) in dichloromethane (60 ml) at 30 °C. After reacting for 12 h, the crude solutions were rotary evaporated and purified by recrystallization from methanol to yield compounds **3a-3g**. It is worth noting that the pure 1-(tert-butyl)-1-ethyl-3-(4-nitrophenyl)thiourea (**3a**) was not obtained under ambient condition owing to the high dynamicity at room temperature (Supplementary Fig. 1a).

1-(4-nitrophenyl)-3-phenylthiourea (**3b**): FTIR (KBr, Supplementary Fig. 1b1): $\nu_{N\text{-}H}$ (3457 cm^{-1}), $\nu_{C\text{-}H}$ (2831-3105 cm^{-1}), $\nu_{N\text{-}C=S(I)}$ (1530 cm^{-1}), $\nu_{N\text{-}C=S(II)}$ (1328 cm^{-1}), and $\nu_{N\text{-}C=S(III)}$ (1116 cm^{-1}). ^{1}H NMR (500 MHz, DMSO-d_6, Supplementary Fig. 1b2): δ 10.36 (s, 1H), 10.24 (s, 1H), 8.27–8.09 (m, 2H), 7.89–7.75 (m, 2H), 7.53–7.45 (m, 2H), 7.40–7.30 (m, 2H), 7.22–7.09 (m, 1H). ^{13}C NMR (500 MHz, DMSO-d_6 and Supplementary Fig. 1b3): δ 179.81, 146.76, 142.76, 139.43, 129.10, 125.49, 124.85, 124.20, and 122.02. Elemental analysis for $C_{13}H_{11}N_3O_2S$, calculated: C, 57.13; H, 4.06; N, 15.37; S, 11.73; and found: C, 57.25; H, 4.08; N, 15.29; S, 11.67.

1-(4-nitrophenyl)-3-cyclohexylthiourea (**3c**): FTIR (KBr, Supplementary Fig. 1c1): $\nu_{N\text{-}H}$ (3421 cm^{-1}), $\nu_{N\text{-}C=S(I)}$ (1509 cm^{-1}), $\nu_{N\text{-}C=S(II)}$ (1338 cm^{-1}), and $\nu_{N\text{-}C=S(III)}$ (1111 cm^{-1}). ^{1}H NMR (500 MHz, DMSO-d_6, Supplementary Fig. 1c2): δ 9.94 (s, 1H), 8.31–8.01 (m, 3H), 7.98–7.68 (m, 2H), 4.09 (s, 1H), 1.93 (m, 1H), 1.81-0.93 (m, 10H). ^{13}C NMR (500 MHz, DMSO-d_6, Supplementary Fig. 1c3): δ 179.12, 147.03, 142.09, 124.95, 120.58, 52.75, 31.97, 25.58, 24.8. Elemental analysis for $C_{13}H_{17}N_3O_2S$, calculated: C, 55.89; H, 6.13; N, 15.04; S, 11.48; and found: C, 56.02; H, 6.11; N, 15.01; S, 11.39.

1,1-diethyl-3-(4-nitrophenyl)thiourea (**3d**): FTIR (KBr, Supplementary Fig. 1d1): $\nu_{N\text{-}H}$ (3367 cm^{-1}), $N_{C\text{-}H}$ (2979 cm^{-1}, 2930 cm^{-1}), $\nu_{N\text{-}C=S(I)}$ (1500 cm^{-1}), $\nu_{N\text{-}C=S(II)}$ (1330 cm^{-1}), and $\nu_{N\text{-}C=S(III)}$ (1108 cm^{-1}). ^{1}H NMR (400 MHz, DMSO-d6, Supplementary Fig. 1d2): δ 9.74–8.99 (m, 1H), 8.47–7.88 (m, 2H), 7.85–7.36 (m, 2H), 4.15–3.55 (m, 4H), 1.49–0.91 (m, 6H). ^{13}C NMR (400 MHz, DMSO-d6, and Supplementary Fig. 1d3): δ 179.77, 148.33, 142.81, 124.24, 45.83, and 13.10. Elemental analysis for $C_{11}H_{15}N_3O_2S$, calculated: C, 52.15; H, 5.97; N, 16.59; S, 12.66; and found: C, 52.15; H, 5.97; N, 16.59; S, 12.66.

1-(4-nitrophenyl)-3-propylthiourea (**3e**): FTIR (KBr, Supplementary Fig. 1e1): $\nu_{N\text{-}H}$ (3217 cm^{-1}), $\nu_{N\text{-}C=S(I)}$ (1512 cm^{-1}), $\nu_{N\text{-}C=S(II)}$ (1342 cm^{-1}), and $\nu_{N\text{-}C=S(III)}$ (1108 cm^{-1}). ^{1}H NMR (400 MHz, $CDCl_3$, Supplementary Fig. 1e2): δ 8.48 (s, 1H), 8.24 (dd, J = 21.4, 9.3 Hz, 2H), 7.51 (dd, J = 41.8, 11.5 Hz, 2H), 6.47 (s, 1H), 3.64 (t, J = 6.7 Hz, 2H), 1.87–1.57 (m, 2H), 1.14–0.87 (m, 3H). ^{13}C NMR (400 MHz, $CDCl_3$, Supplementary Fig. 1e3): δ 180.10, 144.41, 143.15, 125.62,

122.49, 47.34, 22.05, and 11.46. Elemental analysis for $C_{10}H_{13}N_3O_2S$, calculated: C, 50.19; H, 5.48; N, 17.56; S, 13.40; and found: C, 50.21; H, 5.62; N, 17.73; S, 13.03.

1-ethyl-1-methyl-3-(4-nitrophenyl)thiourea (**3f**): FTIR (KBr, Supplementary Fig. 1f1): $\nu_{N\text{-}H}$ (3214 cm^{-1}), $N_{C\text{-}H}$ (2966 cm^{-1}, 2923 cm^{-1}), $\nu_{N\text{-}C=S(I)}$ (1511 cm^{-1}), $\nu_{N\text{-}C=S(II)}$ (1346 cm^{-1}), and $\nu_{N\text{-}C=S(III)}$ (1113 cm^{-1}). ^1H NMR (500 MHz, $CDCl_3$, Supplementary Fig. 1f2) δ 8.20–8.11 (m, 2H), 7.53–7.44 (m, 2H), 7.31 (s, 1H), 3.89 (q, J = 7.1 Hz, 2H), 3.28 (s, 3H), 1.41–1.20 (m, 3H). ^{13}C NMR (500 MHz, $CDCl_3$, Supplementary Fig. 1f3): δ 180.54, 145.86, 143.72, 124.46, 122.88, 49.05, 38.41, and 12.03. Elemental analysis for $C_{10}H_{13}N_3O_2S$, calculated: C, 50.19; H, 5.48; N, 17.56; S, 13.40; and found: C, 50.55; H, 5.43; N, 17.77; S, 13.29.

1-(2-hydroxyethyl)-3-(4-nitrophenyl)thiourea (**3g**): FTIR (KBr, Supplementary Fig. 1g1): $\nu_{O\text{-}H}$ (3400 cm^{-1}), $\nu_{N\text{-}H}$ (3226 cm^{-1}), $\nu_{N\text{-}C=S(I)}$ (1502 cm^{-1}), $\nu_{N\text{-}C=S(II)}$ (1338 cm^{-1}), and $\nu_{N\text{-}C=S(III)}$ (1114 cm^{-1}). ^1H NMR (500 MHz, DMSO-d_6, Supplementary Fig. 1g2) δ 8.23–8.09 (m, 2H), 7.90 (dd, J = 26.2, 9.1 Hz, 2H), 4.87 (s, 1H), 3.72–3.38 (m, 4H). ^{13}C NMR (500 MHz, DMSO-d_6, Supplementary Fig. 1g3): δ 180.52, 146.91, 142.20, 124.96, 120.66, 59.28, and 46.96. Elemental analysis for $C_9H_{11}N_3O_3S$, calculated: C, 44.80; H, 4.60; N, 17.42; S, 13.29; and found: C, 45.23; H, 4.72; N, 17.49; S, 12.82.

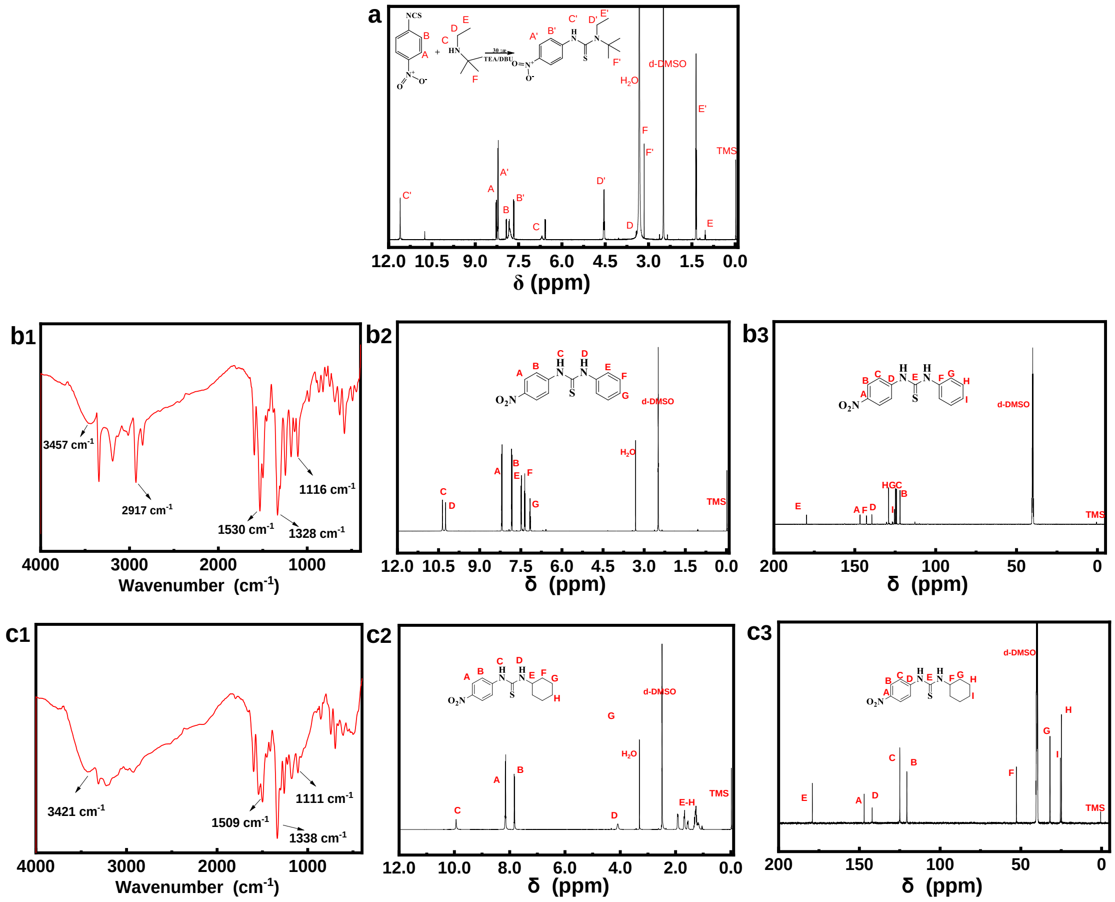

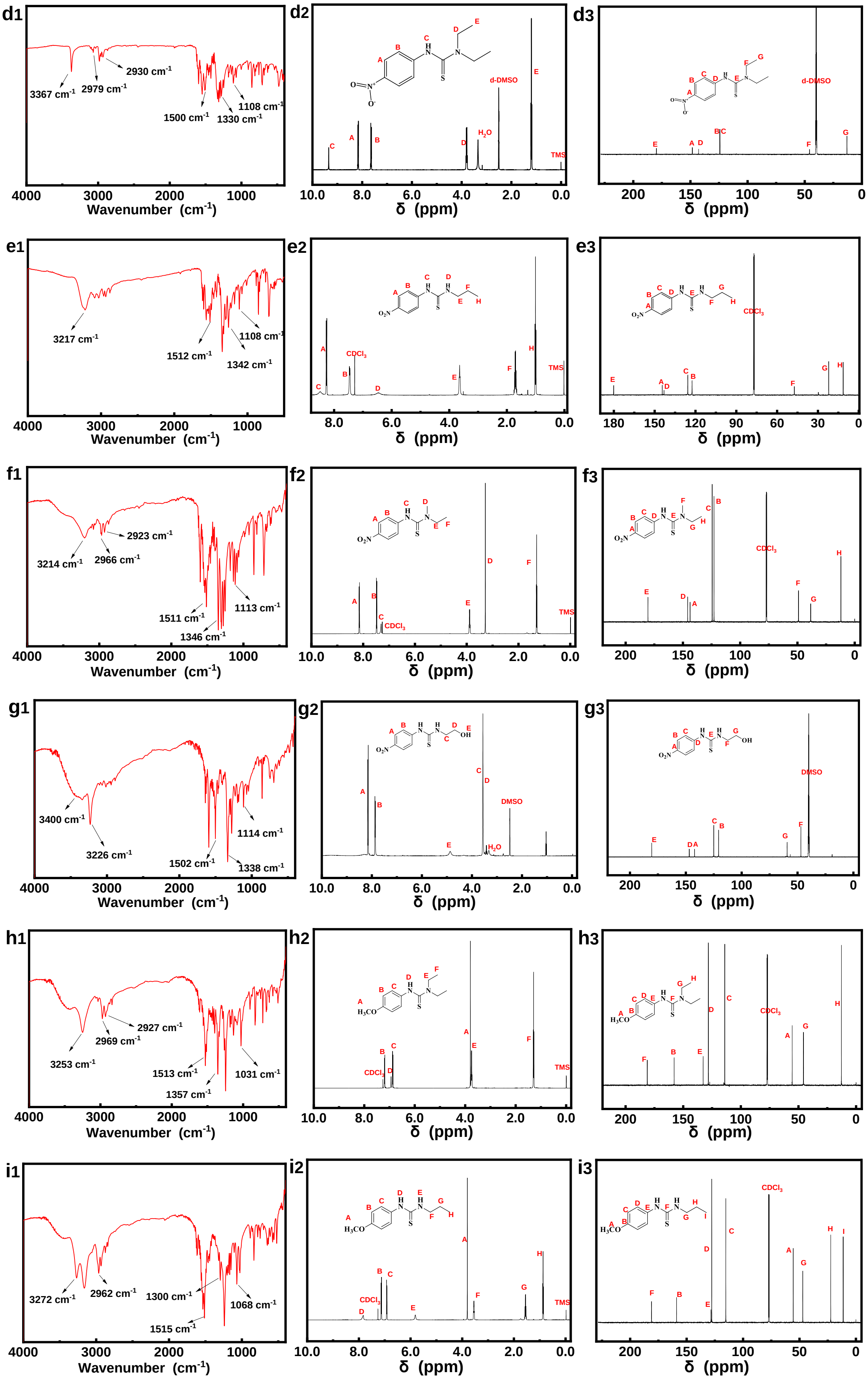
d1
3367 cm-1
2979 cm-1
2930 cm-1
1500 cm-1
1330 cm-1
1108 cm-1
Wavenumber (cm-1)
d2
d-DMSO
H2O
TMS
δ (ppm)
d3
d-DMSO
δ (ppm)
e1
3217 cm-1
1512 cm-1
1342 cm-1
1108 cm-1
Wavenumber (cm-1)
e2
CDCl3
TMS
δ (ppm)
e3
CDCl3
δ (ppm)
f1
3214 cm-1
2966 cm-1
2923 cm-1
1511 cm-1
1346 cm-1
1113 cm-1
Wavenumber (cm-1)
f2
CDCl3
TMS
δ (ppm)
f3
CDCl3
δ (ppm)
g1
3400 cm-1
3226 cm-1
1502 cm-1
1338 cm-1
1114 cm-1
Wavenumber (cm-1)
g2
DMSO
H2O
δ (ppm)
g3
DMSO
δ (ppm)
h1
3253 cm-1
2969 cm-1
2927 cm-1
1513 cm-1
1357 cm-1
1031 cm-1
Wavenumber (cm-1)
h2
CDCl3
TMS
δ (ppm)
h3
CDCl3
δ (ppm)
i1
3272 cm-1
2962 cm-1
1300 cm-1
1515 cm-1
1068 cm-1
Wavenumber (cm-1)
i2
CDCl3
TMS
δ (ppm)
i3
CDCl3
δ (ppm)

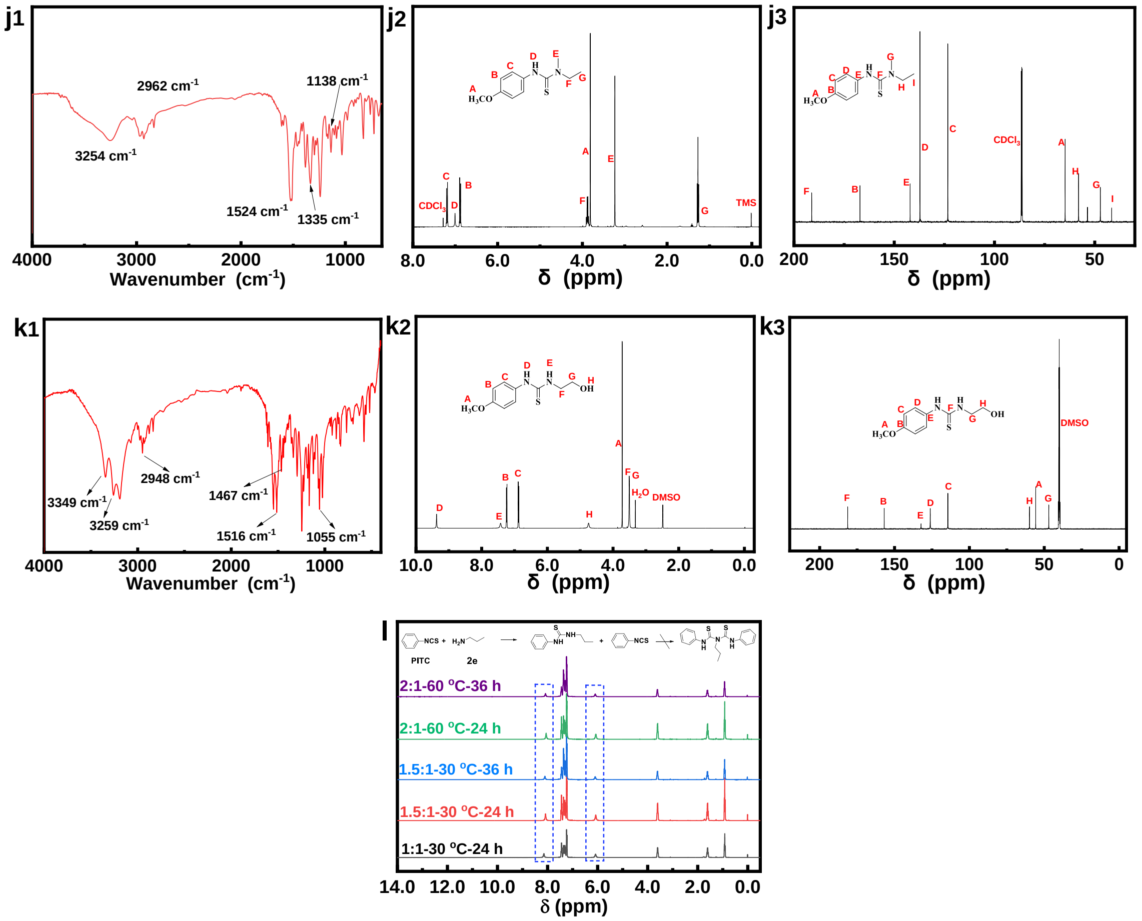

Supplementary Fig. 1 Synthesis and characterization of thiourea molecules. **a** ^{1}H NMR spectrum of **3a**. **b**, **c**, **d**, **e**, **f**, **g**, **h**, **i**, **j**, **k** FTIR, ^{1}H NMR and ^{13}C NMR spectra of (**b1**, **b2**, **b3**) **3b**, (**c1**, **c2**, **c3**) **3c**, (**d1**, **d2**, **d3**) **3d**, (**e1**, **e2**, **e3**) **3e**, (**f1**, **f2**, **f3**) **3f**, (**g1**, **g2**, **g3**) **3g**, (**h1**, **h2**, **h3**) **3′d**, (**i1**, **i2**, **i3**) **3′e**, (**j1**, **j2**, **j3**) **3′f**, and (**k1**, **k2**, **k3**) **3′g**. **l** ^{1}H NMR spectra of the reactions between phenyl isothiocyanate (PITC) and propylamine (**2e**) at different temperatures and molar ratios (PDIT:**2e** = 1:1 or 1.5:1 or 2:1), with constant chemical shifts of δ 8.08 (s, 1H) and δ 6.09 (s, 1H).

Synthesis of Model Thioureas 3′d-3′g. Thiourea compounds **3′d-3′g** were synthesized by mixing **1′** (0.002 mol) with **2d-2g** (0.002 mol) in dichloromethane (60 ml) at 30 °C. After reacting for 12 h, the crude compounds were rotary evaporated and dried, offering the pure thioureas **3′d-3′g**.

1,1-Diethyl-3-(4-methoxyphenyl)thiourea (**3′d**): FTIR (KBr, Supplementary Fig. 1h1): $\nu_{N\text{-}H}$ (3253 cm^{-1}), $\nu_{C\text{-}H}$ (2969 cm^{-1}, 2927 cm^{-1}), $\nu_{N\text{-}C=S(I)}$ (1513 cm^{-1}), $\nu_{N\text{-}C=S(II)}$ (1357 cm^{-1}), and $\nu_{N\text{-}C=S(III)}$ (1031 cm^{-1}). ^{1}H NMR (500 MHz, $CDCl_3$, Supplementary Fig. 1h2) δ 7.26 (s, 1H), 7.19 (d, J = 8.8 Hz, 2H), 6.94 (s, 1H), 6.90–6.85 (m, 2H), 3.81 (d, J = 9.2 Hz, 3H), 3.75 (q, J = 7.1 Hz, 4H), 1.36–1.24 (m, 6H). ^{13}C NMR (500 MHz, $CDCl_3$, Supplementary Fig. 1h3): δ 181.21,

157.86, 132.68, 128.19, 113.95, 55.43, 45.65, and 12.71. Elemental analysis for $C_{12}H_{18}N_2OS$, calculated: C, 60.47 H, 7.61; N, 11.75; S, 13.45; and found: C, 60.69; H, 7.50; N, 11.91; S, 13.24.

1-(4-Methoxyphenyl)-3-propylthiourea (**3′e**): FTIR (KBr, Supplementary Fig. 1i1): $\nu_{N\text{-}H}$ (3272 cm^{-1}), $\nu_{C\text{-}H}$ (2962 cm^{-1}), $\nu_{N\text{-}C=S(I)}$ (1515 cm^{-1}), $\nu_{N\text{-}C=S(II)}$ (1300 cm^{-1}), and $\nu_{N\text{-}C=S(III)}$ (1068 cm^{-1}). ^{1}H NMR (500 MHz, $CDCl_3$, Supplementary Fig. 1i2) δ 7.83 (s, 1H), 7.12 (t, J = 12.8 Hz, 2H), 7.00–6.85 (m, 2H), 5.82 (s, 1H), 3.91–3.74 (s, 3H), 3.55 (dd, J = 12.7, 6.5 Hz, 2H), 1.68–1.44 (m, 2H), 0.96–0.79 (m, 3H). ^{13}C NMR (500 MHz, $CDCl_3$, Supplementary Fig. 1i3): δ 181.09, 158.95, 128.47, 127.77, 115.31, 55.54, 47.08, 22.31, and 11.29. Elemental analysis for $C_{11}H_{16}N_2OS$, calculated: C, 58.90; H, 7.19; N, 12.49; S, 14.29; and found: C, 59.09; H, 7.12; N, 12.71; S, 14.15.

1-Ethyl-1-methyl-3-(4-methoxy)thiourea (**3′f**): FTIR (KBr, Supplementary Fig. 1j1): $\nu_{N\text{-}H}$ (3254 cm^{-1}), $N_{C\text{-}H}$ (2962 cm^{-1}), $\nu_{N\text{-}C=S(I)}$ (1524 cm^{-1}), $\nu_{N\text{-}C=S(II)}$ (1335 cm^{-1}), and $\nu_{N\text{-}C=S(III)}$ (1138 cm^{-1}). ^{1}H NMR (500 MHz, $CDCl_3$, Supplementary Fig. 1j2): δ 7.18 (d, J = 6.0 Hz, 2H), 7.01 (s, J = 27.4 Hz, 1H), 6.96–6.80 (d, 2H), 3.87 (q, J = 7.1 Hz, 2H), 3.81 (s, 3H), 3.22 (s, 3H), 1.26 (t, J = 7.2 Hz, 3H). ^{13}C NMR (500 MHz, $CDCl_3$, Supplementary Fig. 1j3): δ 181.84, 157.75, 132.76, 127.83, 113.96, 55.44, 48.74, 37.87, 12.19. Elemental analysis for $C_{11}H_{16}N_2OS$, calculated: C, 58.90; H, 7.19; N, 12.49; S, 14.29; and found: C, 58.93; H, 7.30; N, 12.54; S, 13.79.

1-(2-Hydroxyethyl)-3-(4-methoxyphenyl)thiourea (**3′g**): FTIR (KBr, Supplementary Fig. 1k1): $\nu_{O\text{-}H}$ (3349 cm^{-1}), $\nu_{N\text{-}H}$ (3259 cm^{-1}), $\nu_{C\text{-}H}$ (2948 cm^{-1}), $\nu_{N\text{-}C=S(I)}$ (1516 cm^{-1}), $\nu_{N\text{-}C=S(II)}$ (1467 cm^{-1}), and $\nu_{N\text{-}C=S(III)}$ (1055 cm^{-1}). ^{1}H NMR (500 MHz, DMSO-d_6, Supplementary Fig. 1k2) δ 9.37 (s, 1H), 7.43 (s, 1H), 7.24 (d, J = 8.9 Hz, 2H), 6.88 (d, J = 8.9 Hz, 2H), 4.75 (s, 1H), 3.69 (d, J = 32.5 Hz, 3H), 3.51 (m, 4H). ^{13}C NMR (500 MHz, DMSO-d_6, Supplementary Fig. 1k3): δ 181.30, 156.90, 132.28, 126.13, 114.36, 59.81, 55.70, and 46.95. Elemental analysis for $C_{10}H_{14}N_2O_2S$, calculated: C, 53.08; H, 6.24; N, 12.38; S, 14.14; and found: C, 53.10; H, 6.07; N, 12.59; S, 14.03.

1.3 Studies of Thermodynamic and Kinetic Properties of the Model Thioureas

1.3.1 Studies of Thermodynamic Properties of the Model Thioureas

Association Equilibrium of 3a. Firstly, **1** (0.101 mol L^{-1}) and **2a** (0.105 mol L^{-1}) were mixed in DMSO-*d6* and quickly transferred to an NMR tube. ^{1}H NMR spectra were collected under 25 °C at selected time intervals until the association/dissociation equilibrium was reached.

Next, the sample was heated for the subsequent variable-temperature experiments (at 35 °C and 55 °C). Finally, the association constants of **3a** at 25 °C, 35 °C and 55 °C were obtained from Supplementary Equation (1) as shown in Supplementary Fig. 2a, and the equilibrium concentrations of **1**, **2a**, and **3a** (i.e. $[\mathbf{1}]_{eq}$, $[\mathbf{2a}]_{eq}$, and $[\mathbf{3a}]_{eq}$) were found not change once the equilibrium was reached.

According to $K_d = 1/K_b$ derived from Supplementary Equation (1) and (2), the dissociation parameters were calculated from the association process. Hence the enthalpy, ΔH_d, and entropy, ΔS_d, of the dissociation reaction can be estimated from van't Hoff equation (Supplementary Equation (3)):

$$K_b = \frac{[\mathbf{3}]_{eq}}{[\mathbf{1}]_{eq}\,[\mathbf{2}]_{eq}} \quad \text{Supplementary Equation (1)}$$

$$K_d = \frac{[\mathbf{1}]_{eq}\,[\mathbf{2}]_{eq}}{[\mathbf{3}]_{eq}} \quad \text{Supplementary Equation (2)}$$

$$\ln K = -\frac{\Delta H}{RT} + \frac{\Delta S}{R} \quad \text{Supplementary Equation (3)}$$

Measurement of Dissociation Temperature, T_d, of the Thiourea Compounds. Here in this work, T_d represents the temperature at which the signals of the dissociative products are able to be detected by in-situ variable-temperature ^{1}H NMR within 30 min. To measure T_d of the thiourea compounds, the dissociation experiments were performed in ^{1}H NMR spectroscope from low to high temperatures at an interval of 5 °C, and the dissociative signals were collected in real-time.

Dissociation Equilibriums of 3b, 3c, 3d and 3e. 3b (0.0646 mol L^{-1}) was dissolved by DMSO-d_6 in an NMR tube, and then the solution was heated to 30 °C for 12 h for pre-equilibrium. Next, in-situ variable-temperature ^{1}H NMR spectra were recorded to study the dissociation of **3b** at 40 °C. The proton spectra were collected at selected time intervals until the dissociation reaction reached equilibrium. Afterwards, the sample was heated to 60 °C at an interval of 5 °C. Finally, the dissociation constants of **3b** were obtained from Supplementary Equation (2) (Supplementary Fig. 2b), while the values of ΔH_d and ΔS_d of the dissociation reactions were calculated from van't Hoff equation (Supplementary Equation (3)). The dissociation studies of **3c**, **3d**, **3e** followed the say way as **3b**. Slightly differently, **3c**, **3d** and **3e** were heated to 80 °C

for 12 h for pre-equilibrium, and the variable-temperature experiments of **3c** (0.0597 mol·L^{-1}, Supplementary Fig. 2c), **3d** (0.075 mol·L^{-1}, Supplementary Fig. 2d), and **3e** (0.0697 mol·L^{-1}, Supplementary Fig. 2e) were carried out at 90–120 °C, 100–125 °C and 100–125 °C, respectively.

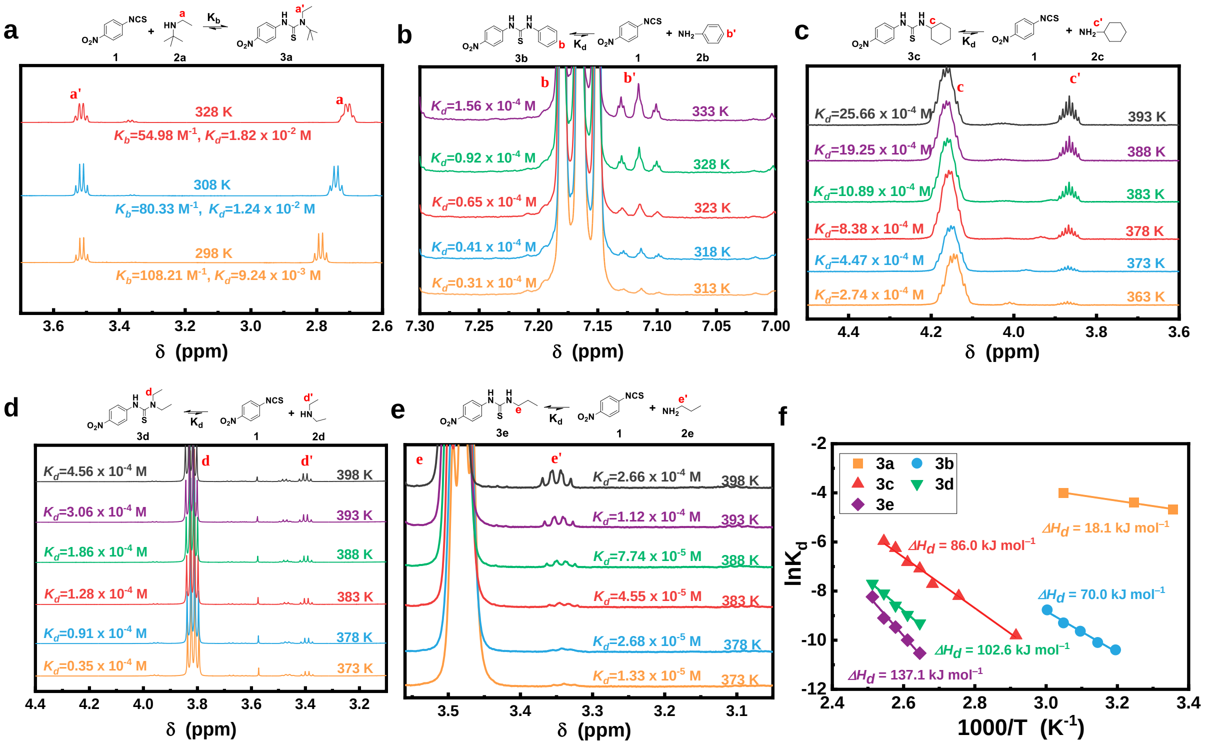

Supplementary Fig. 2 Association/dissociation equilibrium of model thioureas. a Thermodynamic binding equilibrium after mixing **1** and **2a** at different temperatures. **b**, **c**, **d**, **e**, **f** Thermodynamic dissociation equilibria of (**b**) **3b**, (**c**) **3c**, (**d**) **3d** and (**e**) **3e** at different temperatures. **f** Van't Hoff plots of the dissociation reactions of **3a**–**3e**.

1.3.2 Studies of Kinetic Properties of the Model Thioureas

Association Rate of 3a. 1 (0.101 mol L^{-1}) and **2a** (0.105 mol L^{-1}) were mixed in DMSO-d_6. Then, in-situ variable-temperature ^{1}H NMR spectra were immediately collected at selected time intervals under 25 °C. Afterwards, the same processes were carried out at 45 °C and 55 °C, respectively. The rate at every temperature (Supplementary Fig. 3a) is supposed to conform to the first-order kinetics estimated from:

$$\ln(\frac{C_t}{C_0}) = kt \quad \text{Supplementary Equation (4)}$$

where k, t, C_t and C_0 were the rate constant, reaction time, real-time and initial concentrations, respectively.

Furthermore, the association activation energy was estimated to be 20.1 kJ mol^{-1} by fitting the reaction rate constant according to Arrhenius equation (Supplementary Equation (5)):

$$\ln k = -\frac{E_a}{RT} + C \quad \text{Supplementary Equation (5)}$$

The dissociation rate was calculated from the association rate and association equilibrium constant according to $k_d = k_b/K_b$, and the dissociation activation energy was found to be 38.6 kJ mol^{-1} by fitting the dissociation rate constant according to Arrhenius equation (Supplementary Equation (5)).

Dissociation Rates of 3b, 3c, 3d and 3e. 3b was dissolved in DMSO-d_6, and then in-situ variable-temperature ^{1}H NMR spectra were firstly collected at short time intervals under 40 °C. Afterwards, the same processes were carried out at 50, 55 and 60 °C, respectively. The rate at every temperature (Supplementary Fig. 3b) conformed to the first-order kinetics as estimated from Supplementary Equation (4). Furthermore, the dissociation activation energy was calculated by fitting the reaction rate constant according to Arrhenius equation (Supplementary Equation (5)). The dissociation rates of **3c**, **3d** and **3e** were determined following the method of **3b**. Slightly differently, the measurements of **3c** (Supplementary Fig. 3c) were carried out at 100, 105, 110, 115 and 120 °C. The measurements of **3d** (Supplementary Fig. 3d) were carried out at 100, 110, 115, 120 and 125 °C, and those of **3e** (Supplementary Fig. 3e) were carried out at 100, 105, 110, 120 and 125 °C.

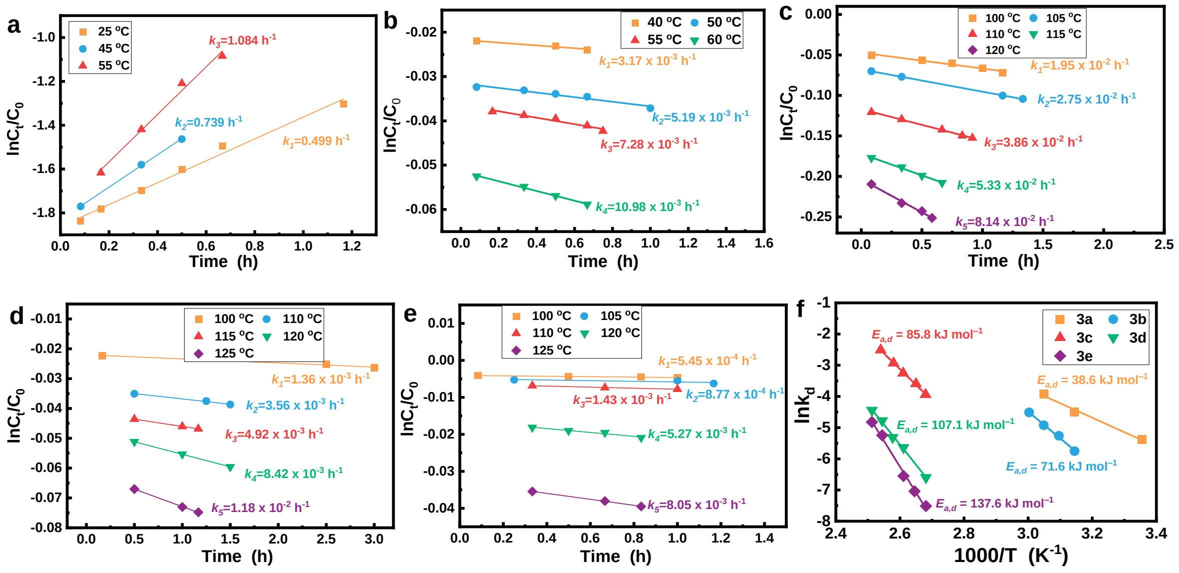

Supplementary Fig. 3 Linear regressions of the experimental data measured at various temperatures according to the first-order kinetics. a The association concentrations were calculated from the bonding product measurements of **3a**. **b**, **c**, **d**, **e** The dissociation concentrations were calculated from the dissociation product measurements of (**b**) **3b**, (**c**) **3c**, (**d**) **3d** and (**e**) **3e**. **f** Arrhenius analyses of the dissociation reactions of **3a**–**3e**.

Supplementary Table 2 Thermodynamic and kinetic parameters of the model thioureas

**Model thioureas**	**3a**	**3b**	**3c**	**3d**	**3e**
T_d **(K)**	298–328	313–333	363–393	373–398	373–398
K_d **(10^{-4} mol L^{-1})**	92.40–181.90	0.31–1.56	2.74–25.66	0.35–4.56	0.13–2.66
ΔH_d **(kJ mol^{-1})**	18.05	70.03	86.01	102.59	137.13
ΔS_d **(J mol^{-1} K^{-1})**	21.82	136.79	168.55	193.58	274.87
T_d **(K)**	298–328	318–343	373–393	373–398	373–398
k_d **(10^{-3} h^{-1})**	4.61–19.72	3.17–10.58	19.5–81.4	1.36–11.78	0.55–8.05
$E_{a,d}$ **(kJ mol^{-1})**	38.6	71.6	85.8	107.1	137.6

1.3.3 Exchange Kinetics Studies of the Model Thioureas

Study of Exchange Reactions between Thiourea and Amine (3e + 2d, 3d + 2e, 3d + 2f, 3d + 2b). 3e (0.04 mmol) and **2d** (0.08 mmol) were mixed in $CDCl_3$ (0.6 ml), and the mixture was heated to 60 °C. In-situ variable-temperature ^1H NMR spectra were collected at selected time intervals. Furthermore, the exchange processes at different temperatures (70 °C, 80 °C and 90 °C, Supplementary Figs. 4a and 4b) were also measured. The exchange of **3e** and **2d** could occur at 60 °C, which conformed to the first-order kinetics (Supplementary Equation (4)). Furthermore, the corresponding exchange activation energy can be estimated by fitting the reaction rate constant according to Arrhenius equation (Supplementary Equation (5)). The exchange reactions of **3d** and **2e** (Supplementary Figs. 4c and 4d), **3d** and **2f** (Supplementary Figs. 4e and 4f), **3d** and **2b** (Supplementary Fig. 5) were also measured by the above approach. The exchange reaction of **3d** with **2e** (or **3d** with **2f**) could occur at 35 °C, and the exchange processes at 40 °C, 45 °C, 50 °C and 55 °C were also recorded. On the other hand, the exchange reaction of **3d** with **2b** can hardly occur at 35–55 °C after 40 min (Supplementary Fig. 5). Even at 60 °C, insignificant exchange signals were detected.

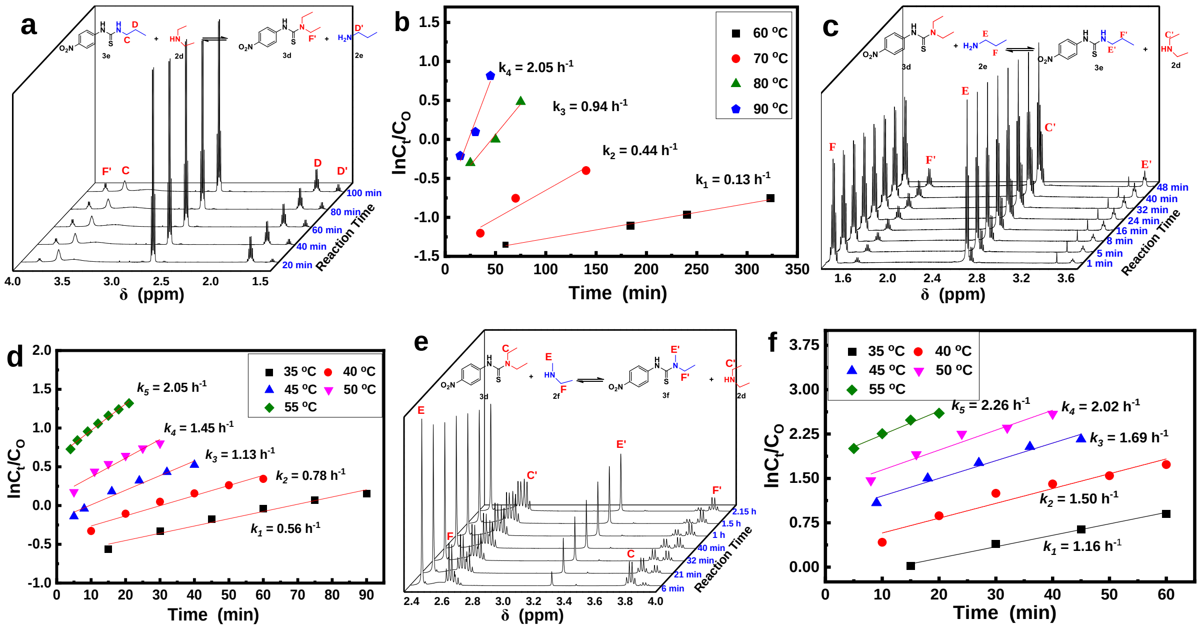

Supplementary Fig. 4 Exchange reactions between thiourea and amine. a, b ^{1}H NMR spectra (**a**) of the exchange between **3e** and **2d** for different durations at 90 °C, and the corresponding linear regressions (**b**) of the data according to the first-order kinetics at various temperatures. **c, d** ^{1}H NMR spectra (**c**) of the exchange between **3d** and **2e** for different durations at 45 °C, and the corresponding linear regressions (**d**) of the data according to the first-order kinetics at different temperatures. **e, f** ^{1}H NMR spectra (**e**) of the exchange between **3d** and **2f** for different durations at 50 °C, and the corresponding linear regressions (**f**) of the data according to the first-order kinetics at various temperatures.

Supplementary Table 3 Kinetic parameters of the exchange reactions of thioureas calculated from the ^{1}H NMR and HPLC results

Materials	3d + 2b	3d + 2f	3d + 2e	3e + 2d	3d + 3'f	3e + 3'g
k_e (h^{-1})	308–328 K	308–328 K	308–328 K	333–363 K	318–343 K	353–393 K
	NA	1.16–2.26	0.56–2.05	0.13–2.05	0.011–0.114	0.0018–0.056
$E_{a,e}$ (kJ mol^{-1})	NA	29.8	53.9	90.2	85.7	99.2

Note: k_e and $E_{a,e}$ were estimated from the first-order kinetics (Supplementary Equation (4)) and Arrhenius equation (Supplementary Equation (5)), respectively. NA: not available.

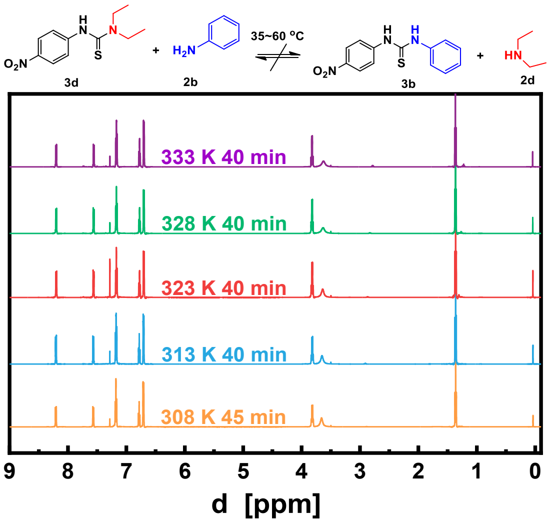

Supplementary Fig. 5 ^{1}H NMR spectra of the exchange reactions between **3d** and **2b** at different temperatures for 40 or 45 min.

Study of Exchange Reactions between Thioureas (3d + 3′f, 3e + 3′g). The mixture of **3d** and **3′f** was heated in a vacuum oven at different temperatures (45–70 °C). The aliquots were periodically taken out and diluted by MeCN (1×10^{-3} mol L^{-1}), and analyzed by high performance liquid chromatograph (HPLC) equipped with a UV detector (254 nm). The eluent was a mixed solutions of MeCN/MeOH (volume ratio = 1:1).

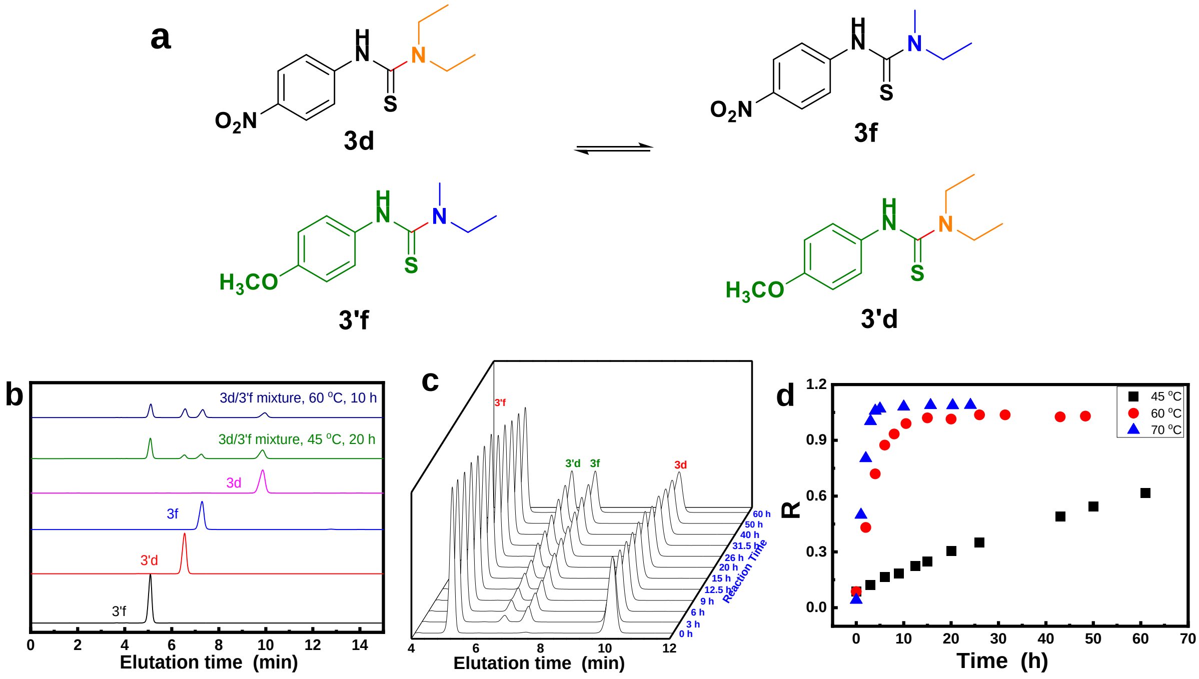

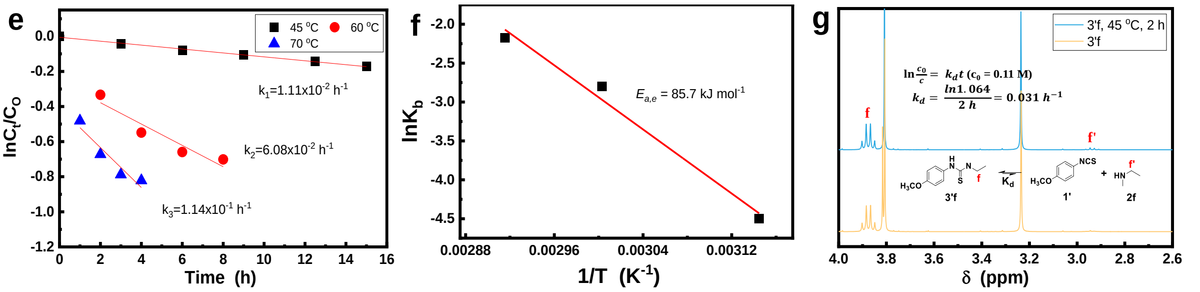

Supplementary Fig. 6 Dynamic exchange of 3d and 3'f. a The sketch showing the exchange reaction. **b** The qualitative proof of the exchange process offered by HPLC analysis. **c** HPLC spectra of the exchange between **3d** and **3'f** for different durations at 45 °C. **d** Chromatographic peak area ratio *R* (*R* = (**3′d** + **3f**)/(**3d** + **3′f**)) as a function of time upon heating the mixture of **3d** and **3'f** at different temperatures. **e** Linear regressions of the data according to the first-order kinetics. **f** Arrhenius analysis of the model exchange reaction. **g** ^{1}H NMR spectra of **3'f** at 45 °C for different times (**3'f** shows a k_d of 0.031 h^{-1} at 45 °C, while 1.36×10^{-3} h^{-1} is achieved for **3d** with bigger steric hindrance (than that of **3f**) at 100 °C). As for the exchange between **3e** and **3′g**, their mixture was heated in a vacuum oven at different temperatures (80–120 °C) in advance. Then, the rest parts of the measurement are the same as those applied for **3d** and **3′f**.

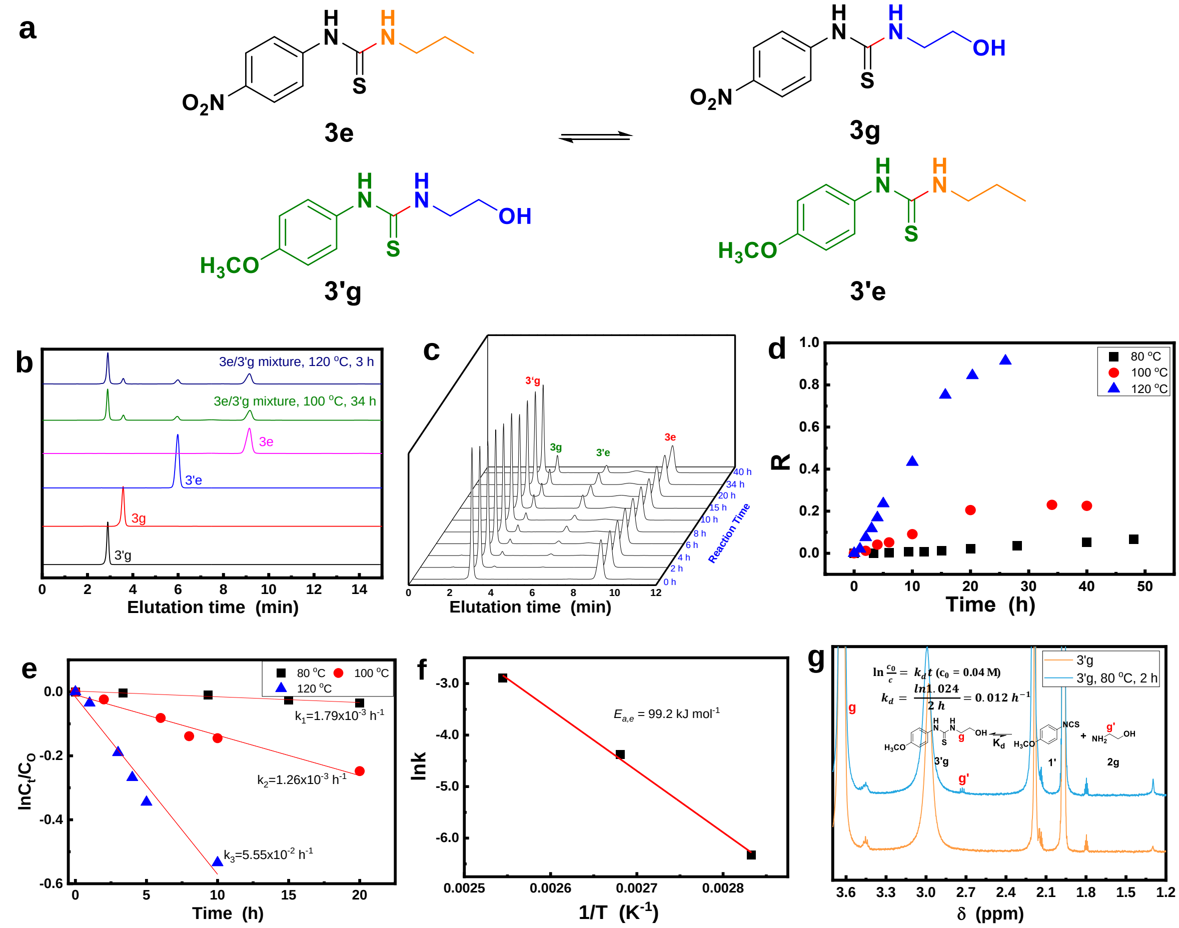

Supplementary Fig. 7 Dynamic exchange of 3e and 3'g. a The sketch showing the exchange reaction. **b** The qualitative proof of exchange process offered by HPLC analysis. **c** HPLC spectra of the exchange between of **3e** and **3'g** at 100 °C for different durations. **d** Chromatographic peak area ratio *R* (*R* = (**3′e** + **3g**)/(**3e** + **3'g**)) as a function of time upon heating the mixture of **3e** and **3'g** at different temperatures. **e** Linear regressions of the data according to the first-order kinetics. **f** Arrhenius analysis of this model exchange reaction. **g** ^{1}H NMR spectra of **3'g** at 80 °C for different times.

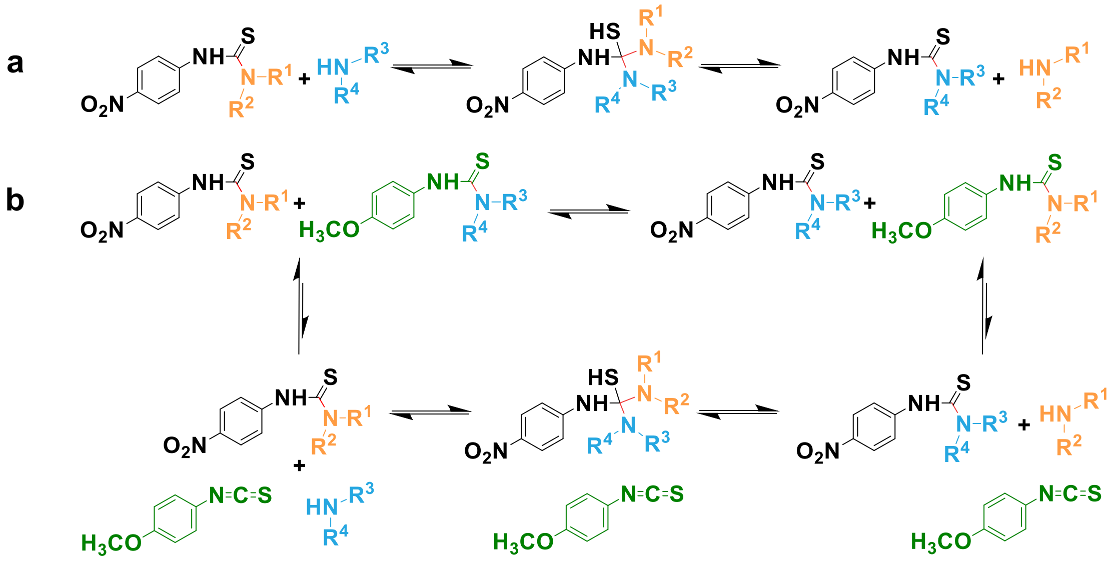

Supplementary Fig. 8 The mechanisms involved in the exchange reactions. a, **b** Intermolecular exchange reactions via (**a**) the associative pathway and (**b**) the combination of dissociative and associative pathways.

1.4 Syntheses of PTUs and the Control

a

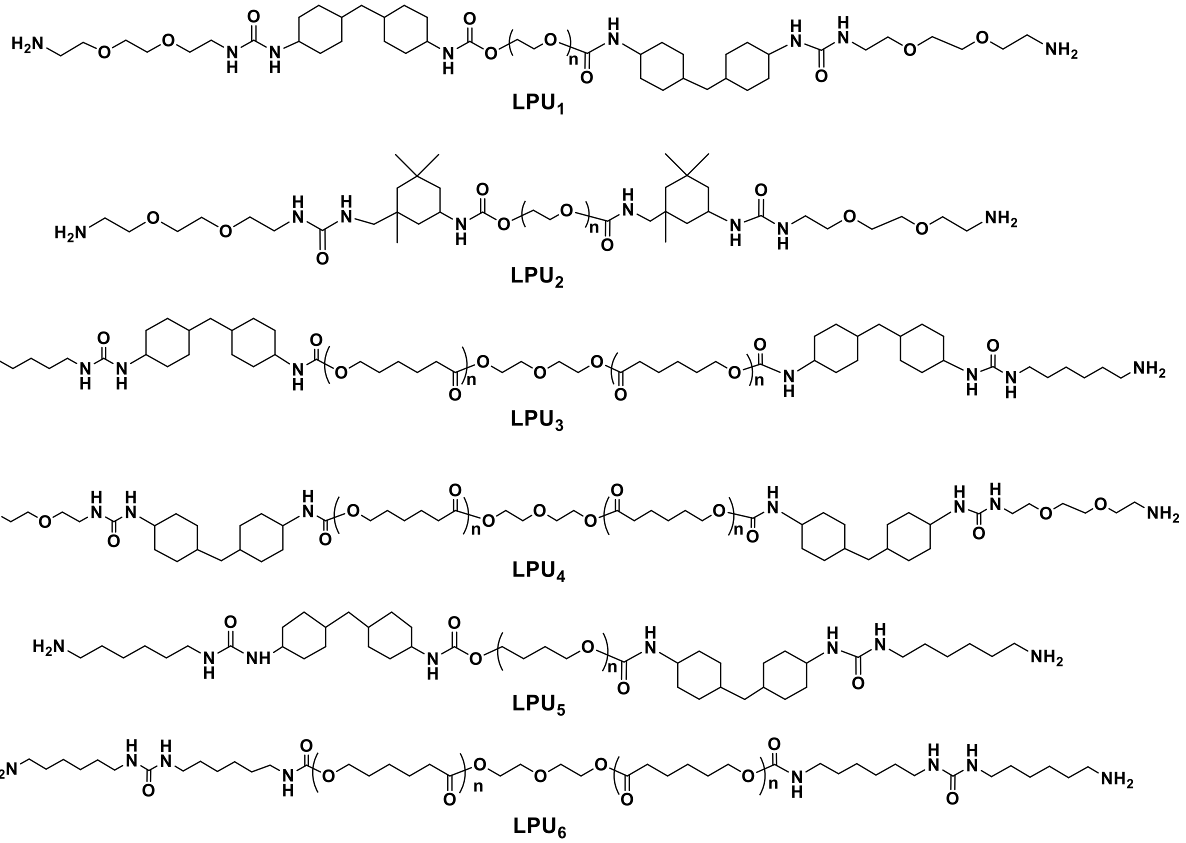

b

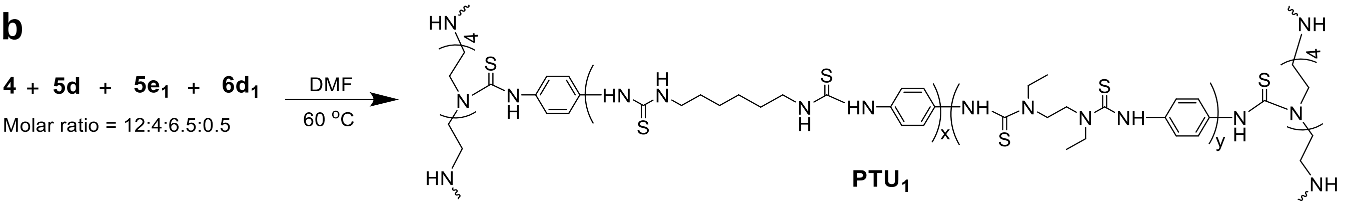

c

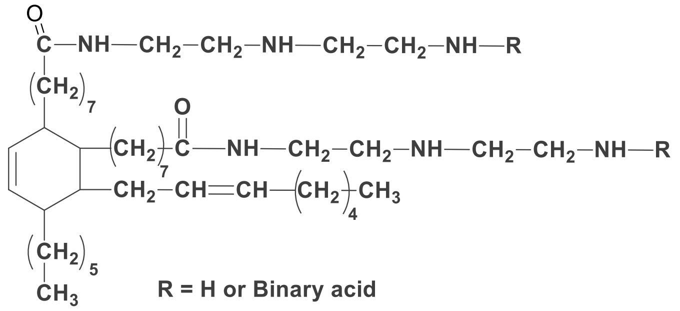

$6d_2$, Polyamide curing agents

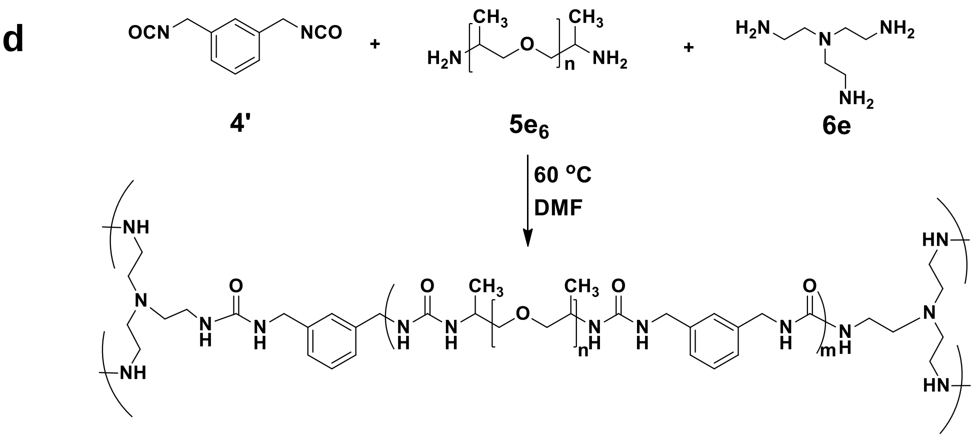

Control polyurethane

Supplementary Fig. 9 Chemical structures of the reactants, and synthesis mechanisms of the crosslinked PTUs and control polyurea. **a** Structures of the amino-terminal linear polyurethanes (**LPU_1–LPU_6**). **b** Synthesis of **PTU_1**. **c** Chemical structure of **$6d_2$**. **d** Synthesis of the control polyurea.

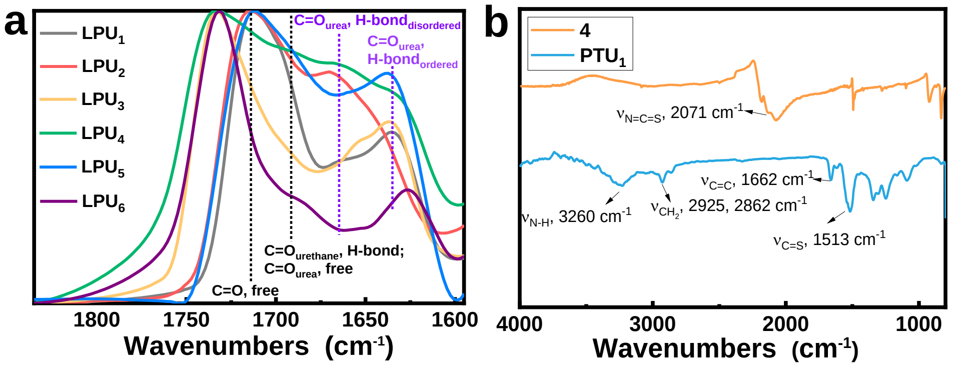

Supplementary Fig. 10 Characterization of chemical structures of linear polyurethanes and PTU. a, **b** FTIR spectra of (**a**) **LPU_1–LPU_6** and (**b**) **PTU_1** and **4**.

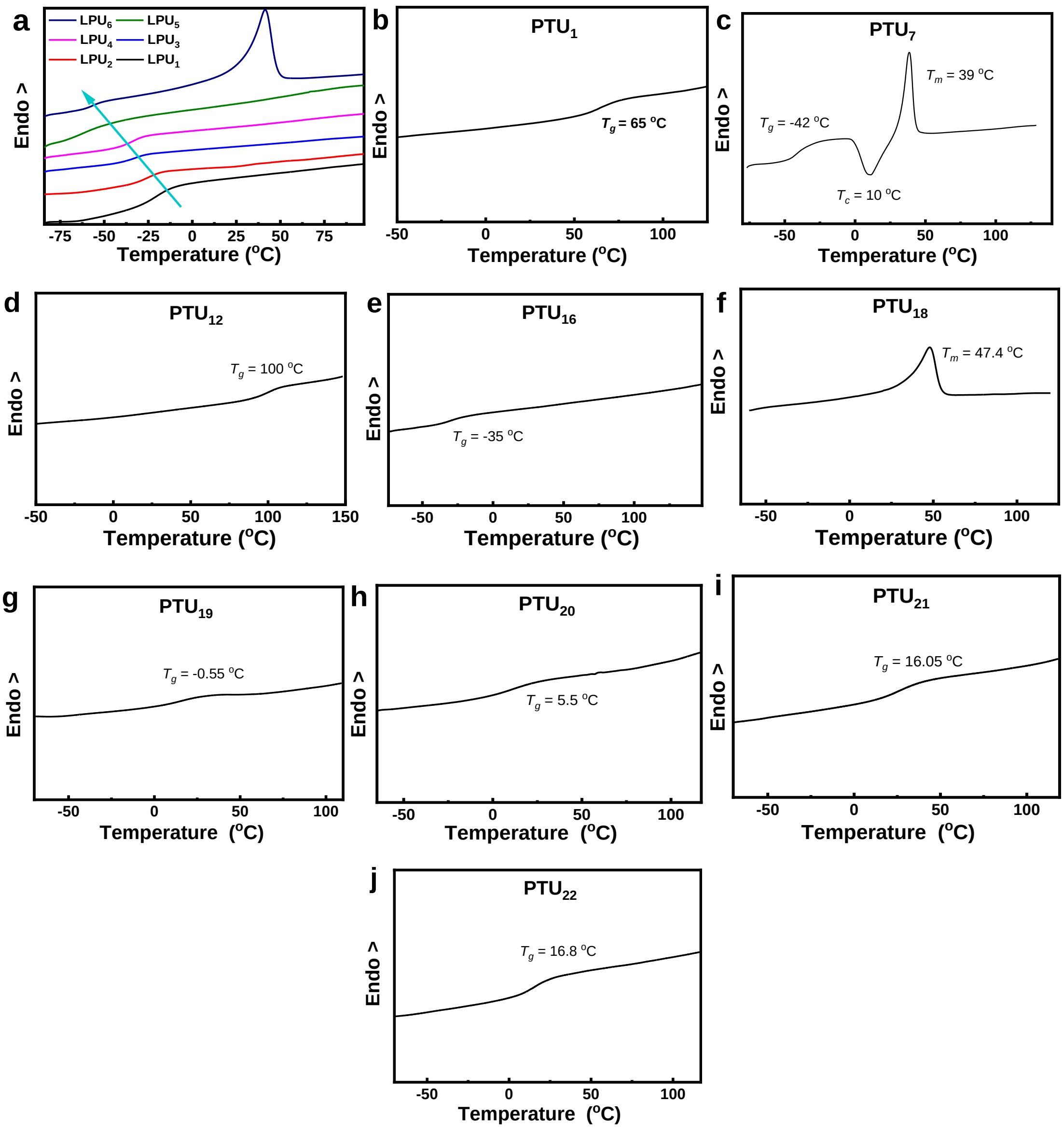

Supplementary Fig. 11 Determination of glass transition temperatures. **a**–**j** DCS heating curves of (**a**) **LPU$_1$**–**LPU$_6$**, (**b**) **PTU$_1$**, (**c**) **PTU$_7$**, (**d**) **PTU$_{12}$**, (**e**) **PTU$_{16}$**, (**f**) **PTU$_{18}$**, (**g**) **PTU$_{19}$**, (**h**) **PTU$_{20}$**, (**i**) **PTU$_{21}$**, and (**j**) **PTU$_{22}$**.

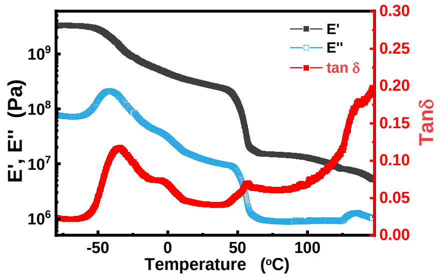

Supplementary Fig. 12 Tan δ, storage modulus, E', and loss modulus, E'', of **PTU$_{18}$** measured between -80 to 140 °C.

Wet Spinning. Bulk **PTU18** was immersed and continuously stirred in DMF at 80 °C, resulting in a uniform solution with a solid content of 10 wt%. Then, the mixture was filtrated and evacuated to thoroughly remove air bubbles. A 30 G flat needle with 0.16 mm inner diameter was used as the spinneret. The spun liquid was squeezed out of the cylinder spinner at room temperature and directly entered a 1 m-long coagulating bath of ethanediol. When precipitation appeared in the coagulation bath, it was stretched, offering continuous **PTU18** filament. The dried filament was further stretched at a drawing ratio of 1 under 60 °C to obtain the target filament.

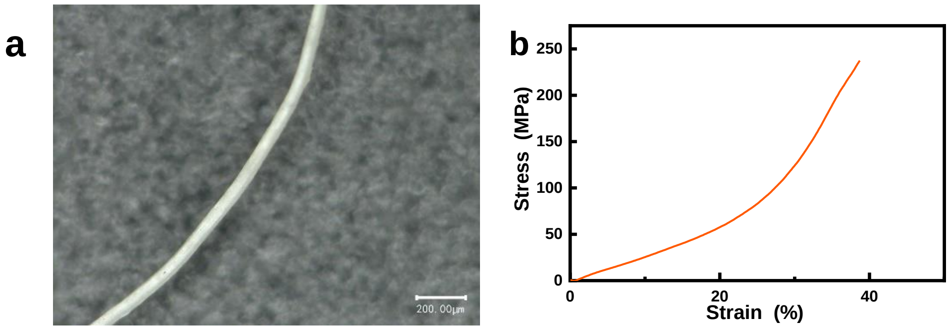

Supplementary Fig. 13 Morphology and mechanical performance of the PTU18 fiber. a, **b** Optical image (**a**) and typical tensile stress-strain curve (**b**) of **PTU18** fiber.

Synthesis of the Control. The DMF solution of m-xylylene diisocyanate (**4′**, XDI, 12 equiv.) was added to a 250 ml three-neck flask under mechanical stirring in nitrogen atmosphere. Then, the dehydrated **5e6** (M_n = 400 g mol^{-1}, 10.5 equiv.) was dropwise incorporated. The mixture was stirred at 60 °C for 6 h, and continued to react with **6e** (1.0 equiv.) for 12 h. At last, the solution was poured into a mold, and cured and dried to obtain the control sample.

1.5 Synthesis of Linear PTUs

Two types of poly(4/5d) were prepared (Supplementary Fig. 14a). Poly(4/5d)-1 was prepared by mixing 1,4-diisothiocyanatobenzene (**4**, 0.005 mol) and N,N'-diethylethylenediamine (**5d,** 0.005 mol) in DMF (60 ml). Meantime, poly(4/5d)-2 was prepared by mixing **4** (0.005 mol) and **5d** (0.0075 mol) in DMF (60 ml). Then, the reaction system was stirred at 60 °C for 12 h. Finally, the solutions were precipitated in methanol, followed by vacuum suction filtration and placed under vacuum at 60 °C for ca. 48 h to obtain the liner PTUs. Linear poly(4/5e1) (Supplementary Fig. 14b) was prepared by the similar approach.

Linear PTU ploy(4/5d): FTIR (KBr, Supplementary Fig. 14c1): ν_{N-H} (3214 cm^{-1}), N_{C-H} (2967 cm^{-1}, 2926 cm^{-1}), $\nu_{N-C=S(I)}$ (1513 cm^{-1}), $\nu_{N-C=S(II)}$ (1350 cm^{-1}), and $\nu_{N-C=S(III)}$ (1120 cm^{-1}). ^{1}H NMR (400 MHz, DMSO-d_6, Supplementary Fig. 14c2) δ 7.20 (s, 4H), 4.41–3.62 (m, 4H), 2.84 (d, J = 29.3 Hz, 4H), 1.46–1.07 (m, 6H). ^{13}C NMR (400 MHz, DMSO-d_6, Supplementary Fig. 14c3): δ 162.78, 127.14, 36.25, 31.23 and 13.07.

Linear PTU ploy(4/5e$_1$): FTIR (KBr, Supplementary Fig. 14d1): ν_{N-H} (3433-3269 cm^{-1}), N_{C-H} (2926 cm^{-1}), $\nu_{N-C=S(I)}$ (1541 cm^{-1}), $\nu_{N-C=S(II)}$ (1339 cm^{-1}), and $\nu_{N-C=S(III)}$ (1074 cm^{-1}). ^{1}H NMR (500 MHz, DMSO-d_6, Supplementary Fig. 14d2) δ 9.36 (s, 1H), 7.87–7.50 (s, 1H), 7.44–7.22 (s, 4H), 3.44 (s, 2H), 1.53 (m, 2H), 1.31 (s, 2H). ^{13}C NMR (500 MHz, DMSO-d_6, Supplementary Fig. 14d3) δ 180.76, 135.90, 123.86, 44.28, 28.98, and 26.70.

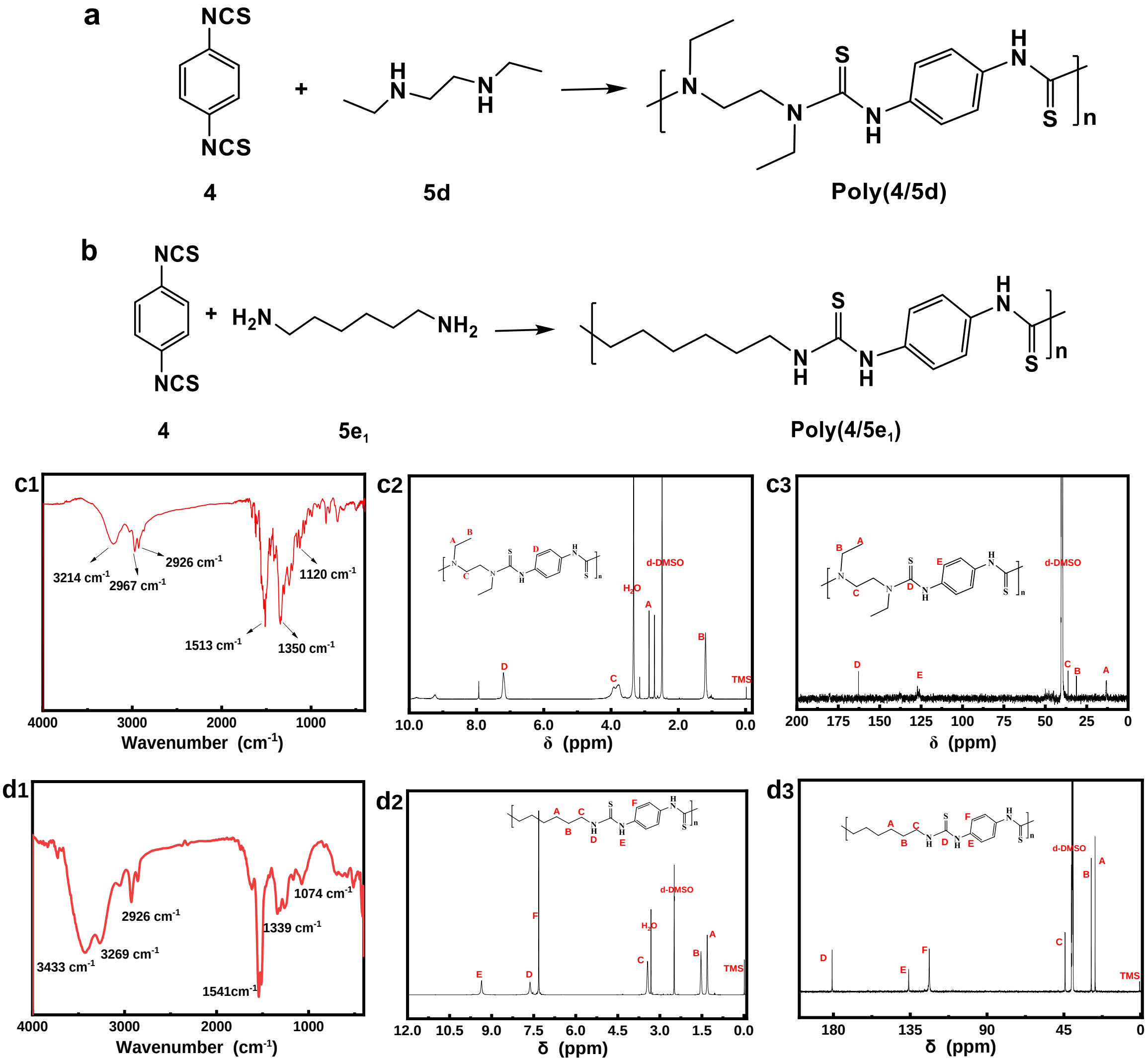

Supplementary Fig. 14 Synthesis of linear PTUs. **a** Poly(4/5d). **b** Poly(4/5e$_1$). **c**, **d** FTIR, ^{1}H NMR and ^{13}C NMR spectra of (**c1**, **c2**, **c3**) poly(4/5d) and (**d1**, **d2**, **d3**) poly(4/5e$_1$).

2. Supplementary Figures and Tables

Supplementary Table 4 Physical properties and healing efficiencies of the crosslinked PTUs

**PTUs**	**Thiourea unit**	**Molar ratio of reactants**	**$T_{g, DSC}$ (°C)**	**$T_{5\%}$ (°C)**	**Mechanical properties**			**Healing conditions**	**Healing efficiency (%)**
					***E* (MPa)**	***σ* (MPa)**	***ε* (%)**		
**PTU_1**	**3d**	**4**: **5d**: **$5e_1$**: **$6d_1$** 12: 4: 6.5: 0.5	65	181	1380.14 ± 93.41	26.11 ± 3.83	17.74 ± 6.93	100 °C, 12 h	98
**PTU_2**	**3f**	**4**: **5f**: **$5e_1$**: **$6d_1$** 12: 4: 6.5: 0.5	62	187	53.60 ± 20.60	11.93 ± 1.64	215.49 ± 16.23	100 °C, 12 h	96
**PTU_3**	**3d**	**4**: **5d**: **$5e_1$**: **$6d_2$** 12: 4: 6.5: 3 (Polyamide115)	73	190	657.2 ± 81.94	20.03 ± 1.53	197.05 ± 8.95	100 °C, 12 h	100
**PTU_4**	**3d**	**4**: **5d**: **$5e_1$**: **$6d_2$** 12: 4: 6.5: 3 (Polyamide125)	67	189	820.27 ± 40.11	21.27 ± 2.88	137.34 ± 17.10	100 °C, 12 h	98.2
**PTU_5**	**3d**	**4**: **5d**: **$5e_3$**: **$6d_1$** 12: 4: 6.5: 0.5	50	187	10.71 ± 4.16	13.73 ± 1.09	212.25 ± 23.81	95 °C, 12 h	97
**PTU_6**	**3d**	**4**: **5d**: **$5e_4$**: **$6d_1$** 12: 4: 6.5: 0.5	60	189	33.57 ± 12.32	15.29 ± 1.82	247.20 ± 30.07	90 °C, 12 h	95
**PTU_7**	**3d**	**4**: **5d**: **$5e_5$** (m=30–32): **$6d_1$** 12: 4: 6.5: 0.5	-42	204	25.48 ± 13.01	6.83 ± 0.39	391.53 ± 28.96	90 °C, 12 h	81
**PTU_8**	**3d**	**4**: **5d**: **$5e_6$** (n=5–6): **$6d_1$** 12: 4: 6.5: 0.5	35	196	22.53 ± 5.81	6.14 ± 0.54	523.75 ± 38.10	80 °C, 12 h	98
**PTU_9**	**3d**	**4**: **5d**: **$5e_6$** (n=14–15): **$6d_1$** 12: 4: 6.5: 0.5	-20	286	0.024 ± 0.05	0.71 ± 0.22	442.26 ± 74.63	60 °C, 12 h	100
**PTU_{10}**	**3d, 3c**	**4**: **5d**: **5c**: **$6d_1$** 12: 4: 6.5: 0.5	90	181	1708.36 ± 310.57	53.57 ± 2.94	4.17 ± 0.65	110 °C, 2 MPa, 1 h	94
**PTU_{11}**	**3e**	**4**: **$5e_4$**: **6e** 12: 10.5: 1	31	195	417.11 ± 89.44	26.63 ± 4.48	232.33 ± 53.13	110 °C, 12 h	87
**PTU_{12}**	**3c**	**4**: **5c**: **6e** 12: 10.5: 1	100	192	1971.93 ± 120.50	52.99 ± 2.95	6.89 ± 2.76	120 °C, 2 MPa, 1 h	92
**PTU_{13}**	**3e**	**4**: **$5e_2$**: **6e** 12: 10.5: 1	53	198	398.05 ± 119.46	13.46 ± 0.46	75.80 ± 4.89	110 °C, 12 h	100
**PTU_{14}**	**3b**	**4**: **$5b_1$**: **6e** 12: 10.5: 1	63	171	2089.75 ± 206.53	37.35 ± 4.30	19.33 ± 4.53	80 °C, 12 h	93
**PTU_{15}**	**3b**	**4**: **$5b_2$**: **6e** 12: 10.5: 1	33	178	1338.01 ± 248.57	31.89 ± 2.33	12.57 ± 0.57	80 °C, 12 h	90

**PTU$_{16}$**	**3d**	**4: 5d: LPU$_3$: 6d$_1$** 12: 4: 6.5: 0.5 PCLD, M_n = 1000	-30	223	69.65 ± 24.86	70.03 ± 3.55	390.57 ± 16.26	100 °C, 2 MPa, 1.5 h	96
**PTU$_{17}$**	**3d**	**4: 5d: LPU$_5$: 6d$_1$** 12: 4: 6.5: 0.5 PTMG, M_n = 1000	-47	225	159.04 ± 14.07	56.51 ± 6.34	339.51 ± 39.47	120 °C, 2 MPa, 1 h	91
**PTU$_{18}$**	**3d**	**4: 5d: LPU$_6$: 6d$_1$** 12: 4: 6.5: 0.5 PCLD, M_n = 3000	-42	251	61.94 ± 12.19	36.17 ± 3.17	942.16 ± 88.05	120 °C, 2 MPa, 1 h	87.1
**PTU$_{19}$**	**3a**	**4:5a: LPU$_1$: 6d$_1$** 12:4:6.5:0.5 PEG, M_n = 600	-0.55	200	3.33 ± 0.52	10.27 ± 2.41	712.58 ± 31.41	R.T., 10 MPa, 24 h	100
**PTU$_{20}$**	**3a**	**4: 5a: LPU$_4$: 6d$_1$** 12: 4: 6.5: 0.5 PCLD, M_n = 1000	5.05	239	11.58 ± 2.38	17.91 ± 1.73	667.69 ± 25.11	R.T., 10 MPa, 24 h	100
**PTU$_{21}$**	**3b**	**4: 5b$_1$: LPU$_2$: 6d$_1$** 12: 4: 6.5: 0.5 PEG, M_n = 600	16.05	214	5.36 ± 1.61	10.65 ± 0.98	568.58 ± 44.26	R.T., 10 MPa, 24 h	84
**PTU$_{22}$**	**3b**	**4:5b$_1$: LPU$_2$: 6d$_1$** 12: 2: 8.5: 0.5 PEG, M_n = 400	16.8	225	3.80 ± 0.86	2.40 ± 0.31	1246.33 ± 137.75	R.T., 12 h	100
**PTU$_{23}$**	**3d**	**4: 5d: LPU$_6$: 6d$_1$** 12: 8: 2.5: 0.5 PCLD, M_n = 3000	NA	220	43.17 ± 9.14	54.75 ± 1.51	593.20 ± 18.67	120 °C, 2 MPa, 1 h	90
**Polyurea**	NA	**4′: 5e$_6$** (n = 5–6): **6e** 12: 10.5: 1	15	324	25.47 ± 1.46	4.22 ± 0.29	290.69 ± 23.35	NA	NA

Note: *E*: Young's modulus. *σ*: tensile strength. *ε*: elongation to break. R.T.: room temperature. NA: not available. All materials shown in the table have not been treated by solid-state drawing.

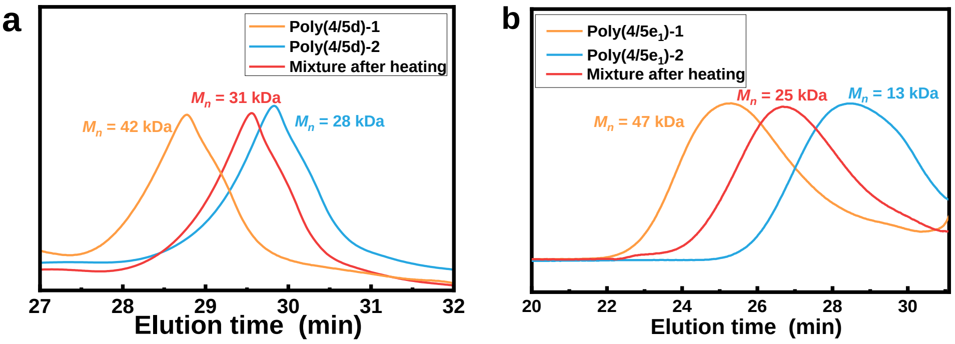

Supplementary Fig. 15 Dynamic exchange of linear PTUs. a GPC curves of poly(4/5d)-1, poly(4/5d)-2 and their mixture after treatment at 60 °C for 12 h. **b** GPC curves of poly(4/5e$_1$)-1 and poly(4/5e$_1$)-2 and their mixture after treatment at 100 °C for 12 h.

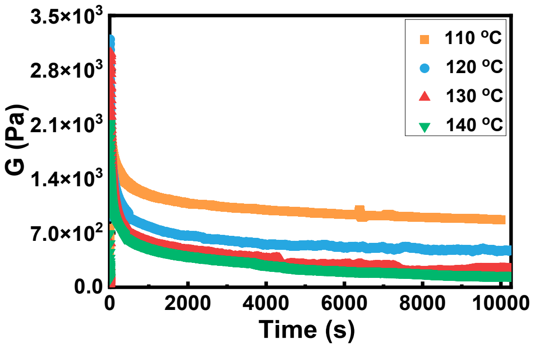

Supplementary Fig. 16 Stress relaxation behaviors of the control polyurea measured at various temperatures.

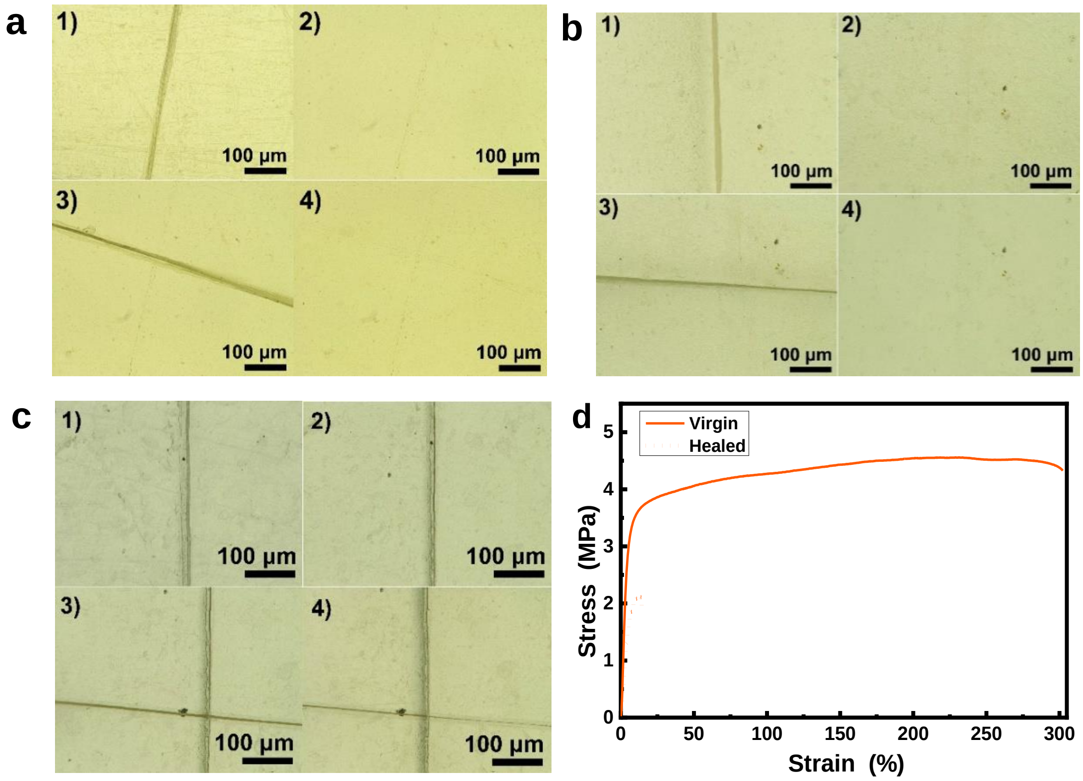

Supplementary Fig. 17 Self-healing performance of crosslinked PTUs and the control polyurea. a, **b**, and **c** Photos showing repeated healing of (**a**) **PTU$_1$**, (**b**) **PTU$_{11}$** and (**c**) control polyurea. (1) The first cut; (2) effect of the first repair; (3) the second cut; (4) effect of the second repair. **d** Typical tensile stress-strain curves of the virgin and healed control polyurea.

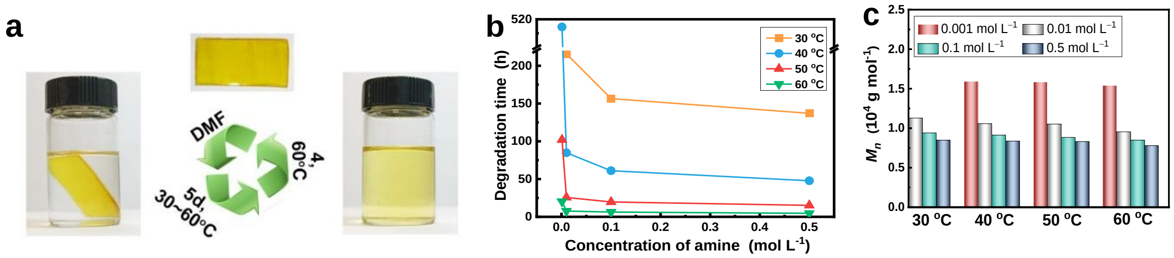

Supplementary Fig. 18 Recycling of crosslinked PTUs. a Photos showing solution-assisted recycling of **PTU1**. **b** Degradation time versus content of added amine measured at different temperatures. **c** M_n of the degraded products obtained at different temperatures.

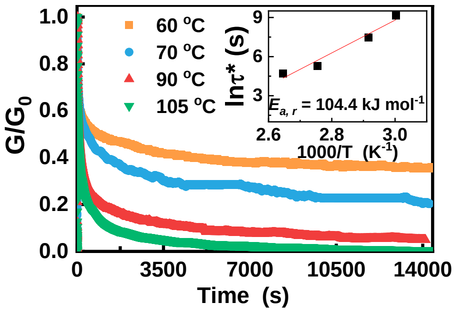

Supplementary Fig. 19 Normalized time dependences of stress relaxation of **PTU18**.

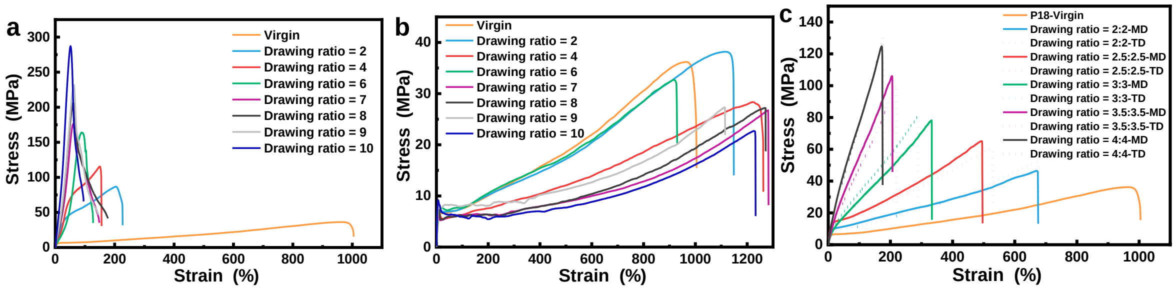

Supplementary Fig. 20 Mechanical performance of the drawn PTUs. **a**, **b** Typical tensile stress-strain curves of original and uniaxially drawn **PTU18** (**a**) along and (**b**) perpendicular to the drawing direction as a function of drawing ratio, respectively. **c** Typical tensile stress-strain curves of biaxially drawn **PTU18** as a function of drawing ratio.

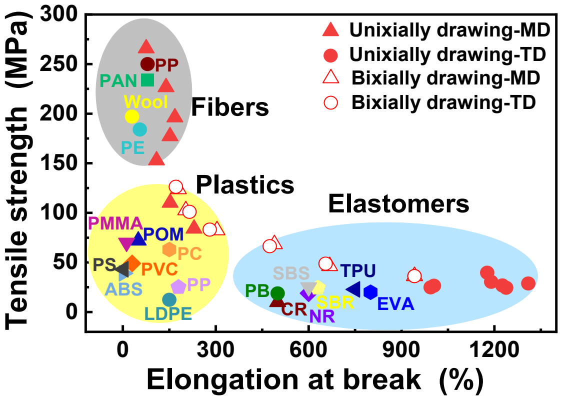

Supplementary Fig. 21 Comparison of the mechanical properties of drawn **PTU18** developed in this work with those of conventional plastics, elastomers and fibers.

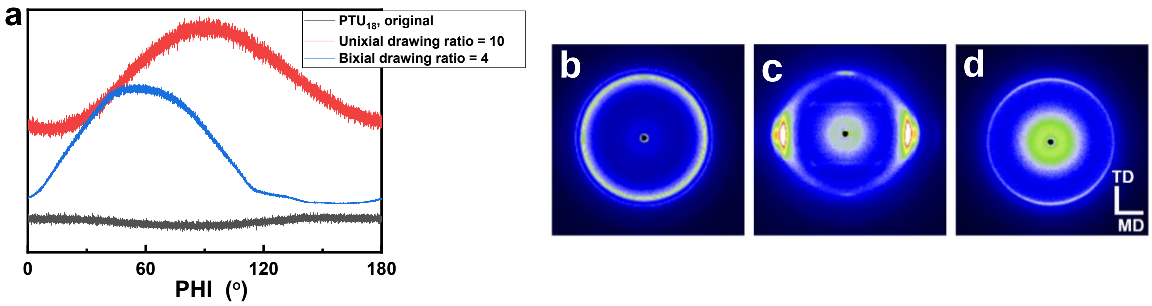

Supplementary Fig. 22 X-ray diffraction measurements. **a** Rotating orientation WXRD patterns of **PTU18**. **b**, **c**, and **d** 2D WXRD patterns of pristine, uniaxially drawn (drawing ratio = 10), and biaxially drawn (drawing ratio = 4) **PTU18**.

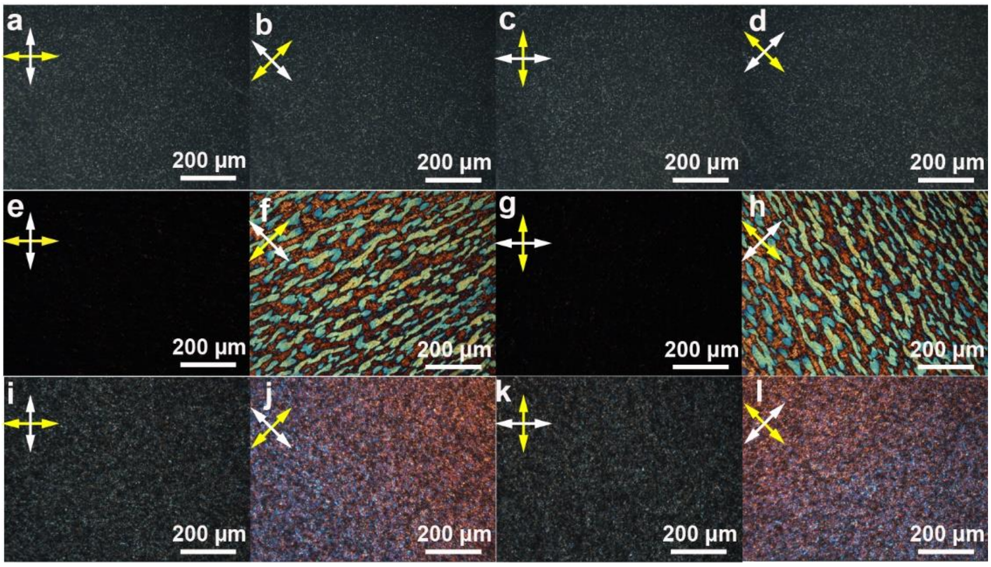

Supplementary Fig. 23 Morphologies observed by orthogonal polarizing microscope. Photos taken by rotating original unstretched (**a**, **b**, **c** and **d**), uniaxially drawn (**e**, **f**, **g** and **h**, drawing ratio = 10) and biaxially drawn (**i**, **j**, **k** and **l**, drawing ratio = 4) **PTU18** under polarized

light. For **a**, **b**, **c**, and **d**, the sample was randomly placed on the stage, and the position was used as the base one of 0°. Then, the stage was rotated 45°, 90° and 135° successively for observation. For **e**, **g**, **i** and **k**, the alignment direction of crystalline regions is parallel to any of the orthogonal polarizers. For **f**, **h**, **j** and **l**, the alignment direction of crystalline regions is 45° with any of the orthogonal polarizers.

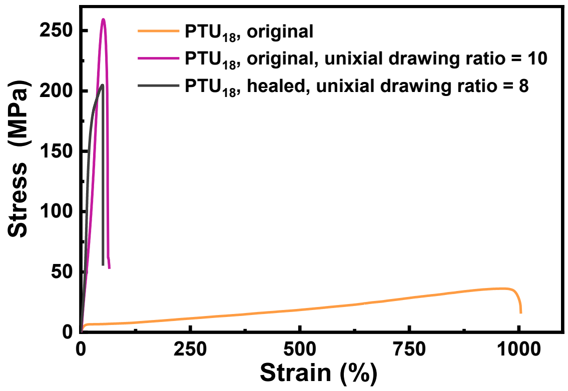

Supplementary Fig. 24 Typical tensile stress-strain curves of original **PTU18**, uniaxially drawn **PTU18** and healed **PTU18**. Note: The healed **PTU18** means the specimen of uniaxially drawn **PTU18** was cut and healed (120 °C, 2 MPa, 1 h), and eventually uniaxially drawn again. The cut unidirectionally stretched **PTU18** was not healed without solid-state drawing, because disorientation would take place during the healing at 120 °C, which is higher than the solid-state drawing temperature (60 °C). As a result, mechanical property of the healed specimen would only be comparable to that of the unstretched one at most, which does not make sense for the present study.

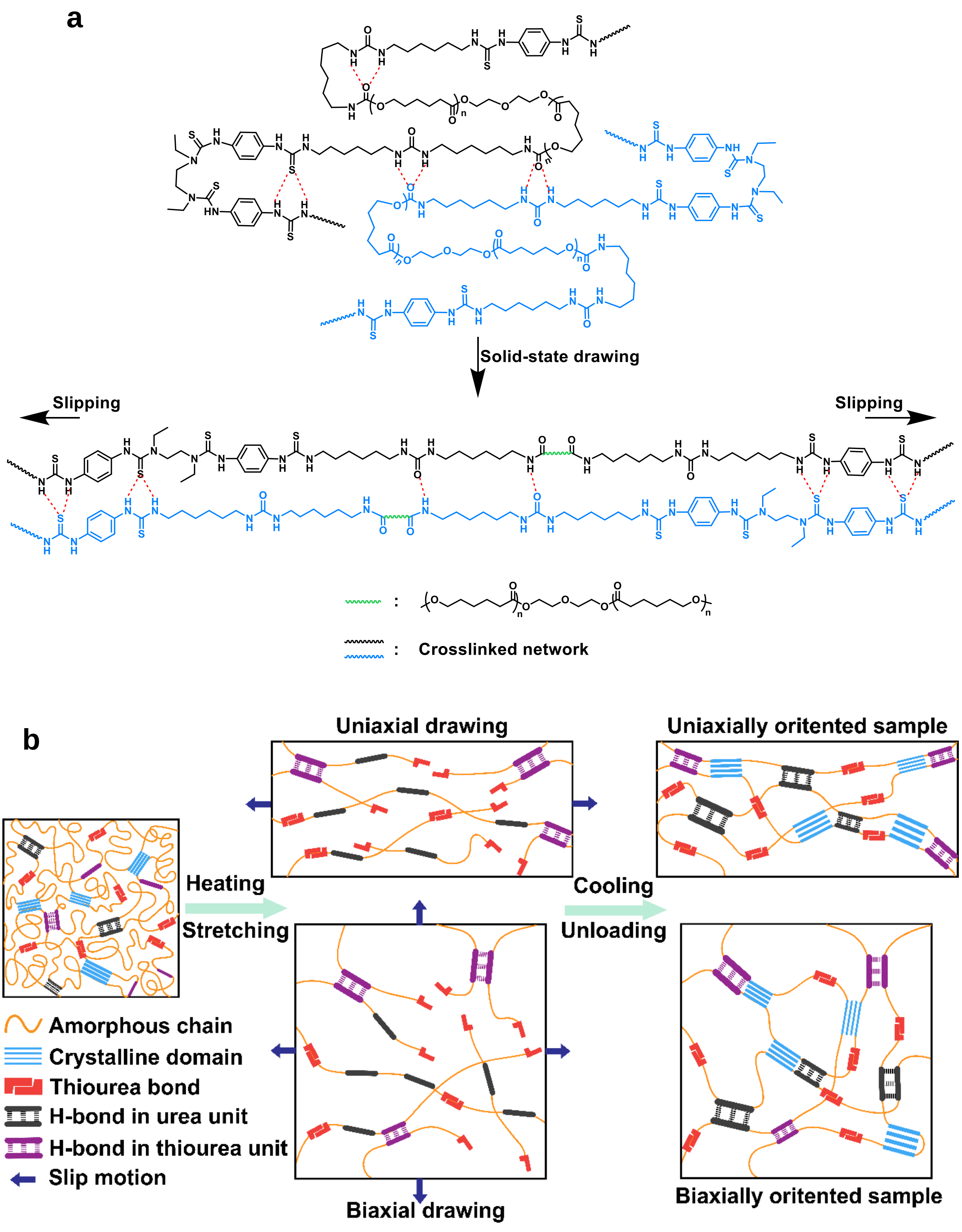
a
Solid-state drawing
Slipping
Slipping
Crosslinked network
b
Uniaxial drawing
Uniaxially oritented sample
Heating
Stretching
Cooling
Unloading
Biaxial drawing
Biaxially oritented sample
Amorphous chain
Crystalline domain
Thiourea bond
H-bond in urea unit
H-bond in thiourea unit
Slip motion

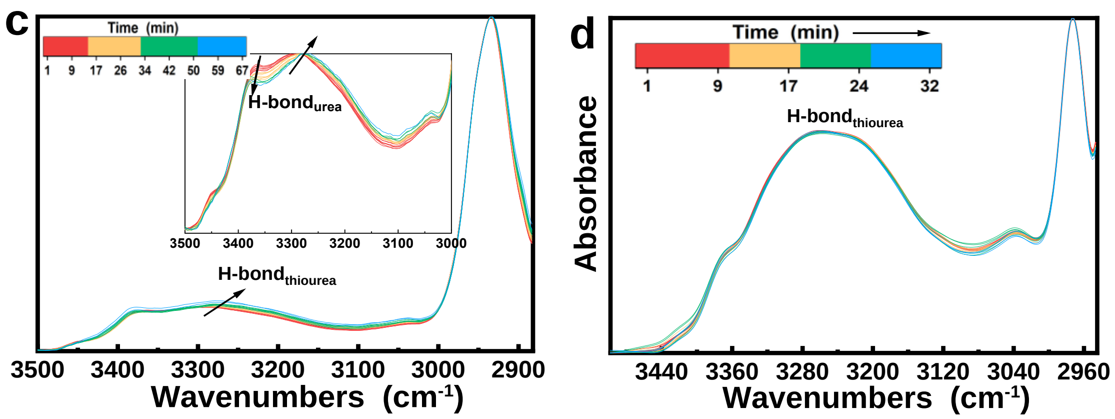

Supplementary Fig. 25 Exploration of the mechanism involved in solid-state drawing. **a** Change of H-bonds in **PTU18** networks during solid-state drawing. **b** Schematic diagram showing microstructural changes of crosslinked PTU during solid-state drawing. **c**, **d** Variation in -NH- peaks quoted from the real-time FTIR spectra of (**c**) **PTU23** and (**d**) **PTU8** under stretching (1 mm min^{-1}, 60 °C).

Supplementary Table 5 Uniaxial orientation degrees of **PTU18** estimated from the rotating orientation WXRD patterns of the samples with 2θ fixed at 21.3° attributed to (110) crystal plane of polycaprolactone segments[2]

Sample	FWHM (°)	Orientation parameter O_{rel}
**Uniaxial drawing ratio = 4**	103.8308	0.423162
**Uniaxial drawing ratio = 6**	88.85352	0.506369
**Uniaxial drawing ratio = 8**	88.72528	0.507082
**Uniaxial drawing ratio = 9**	83.18606	0.537855
**Uniaxial drawing ratio = 10**	82.02321	0.544316

Supplementary Table 6 Mechanical properties along drawing direction of the uniaxially drawn PTUs

Sample	E (MPa)	σ (MPa)	ε (%)	σ/σ_0	M_n of prepolymer (g mol^{-1})
**PTU$_8$, uniaxial drawing ratio = 3^a**	20.90 ± 4.63	6.38 ± 0.61	382.09 ± 30.78	1.04	16184
**PTU$_{12}$, uniaxial drawing ratio = 1^a**	2983.60 ± 113.32	67.20 ± 5.64	5.45 ± 2.82	1.27	47069
**PTU$_{17}$, uniaxial drawing ratio = 5^b**	130.62 ± 22.14	107.11 ± 7.47	95.12 ± 2.09	1.89	147955
**PTU$_{18}$, uniaxial drawing ratio = 10^a**	390.96 ± 9.99	265.88 ± 14.90	65.85 ± 4.09	7.35	200647
**PTU$_{23}$, uniaxial drawing ratio = 3^a**	117.49 ± 23.07	106.14 ± 6.02	141.68 ± 6.65	1.94	142137

Note: ^{a} 60 °C, 1 mm min^{-1}; ^{b} 100 °C, 0.5 mm min^{-1}.

Supplementary References

1. Hammer, B., Hansen, L. B. & Nørskov, J. K. Improved adsorption energetics within density-functional theory using revised Perdew-Burke-Ernzerhof functionals. *Phys. Rev. B* **59**, 7413-7421 (1999).

2. Ji, Y., Liang, K., Shen, X. & Bowlin, G. L. Electrospinning and characterization of chitin nanofibril/polycaprolactone nanocomposite fiber mats. *Carbohydr. Polym.* **101**, 68-74 (2014).
